# The Transformation and Protein Expression of the Edible Mushroom *Stropharia rugosoannulata* Protoplasts by *Agrobacterium-tumefaciens*-Mediated Transformation

**DOI:** 10.3390/jof11090674

**Published:** 2025-09-12

**Authors:** Dongjie Yin, Hairong Xiong

**Affiliations:** College of Life Sciences, South-Central Minzu University, Wuhan 430074, China

**Keywords:** *Agrobacterium-tumefaciens*-mediated transformation, fungal genetics, protein expression, mushroom, protoplast

## Abstract

*Stropharia rugosoannulata* is a cultivated edible mushroom characterized by its nutritional composition and efficient cellulolytic enzymatic systems. However, the lack of genetic tools has significantly impeded the investigation of its molecular mechanisms, severely constraining the study of functional genomic and precision breeding in *S. rugosoannulata*. It was demonstrated in this study that the *Agrobacterium-tumefaciens*-mediated genetic transformation (ATMT) system is applicable for the transformation of *S. rugosoannulata* protoplasts. Through this proposal, we successfully achieved the expression of exogenous genes (*mCherry* gene encoding red fluorescent protein, *hph* gene encoding hygromycin B phosphotransferase, and *GUS* gene encoding β-glucuronidase) and the endogenous mutant gene *SDI* encoding the iron-sulfur protein subunit of succinate dehydrogenase in *S. rugosoannulata*. Furthermore, this study employed endogenous promoters of *GPD* encoding glyceraldehyde-3-phosphate dehydrogenase and *SDI* to enhance transformation efficiency and drive target gene expression. This study establishes the feasibility of ATMT in *S. rugosoannulata* systems, while achieving stable expression of a panel of selectable marker genes and reporter genes critical for genetic research in *S. rugosoannulata*.

## 1. Introduction

*Stropharia rugosoannulata* (*Stropharia rugosoannulata* Farl. ex Murrill), an edible cultivated fungus of Basidiomycota, Agaricales, Strophariaceae, is a straw-rotting species with a wide geographical distribution [1]. In field trials, *S. rugosoannulata* achieved a lignocellulose degradation rate of 40–60% within 90 days when cultivated on rice/wheat straw substrates, significantly enhancing nutrient availability for subsequent crop rotations. As a result of its high yield, *S. rugosoannulata* has become one of the edible fungi recommended by Food and Agriculture Organization of the United Nations (FAO) for cultivation in developing countries, and it also has a large trading volume in the international edible mushroom market [2]. The fruiting body of *S. rugosoannulata* is a kind of high-quality edible fungus with high protein, low fat, and rich carbohydrates [3]. The bioactive components of *S. rugosoannulata* mainly include polysaccharides, lectins, small peptides, and sterols [4]. It exhibits various pharmacological activities, such as immunomodulation, antitumor, anticancer, and antioxidant effects [3,5], and it has preventive effects on hyperglycemia, coronary heart disease [6,7], etc. Zhang et al. isolated a lectin glycoprotein from the fruiting bodies of *S. rugosoannulata*. The experimental results indicated that the lectin exhibited inhibitory activity on the proliferation of hepatocellular carcinoma Hep G2 cells and leukemia L1210 cells while also inhibiting the activity of HIV-1 reverse transcriptase [8]. Other small molecules such as flavonoids and sterols were also shown to possess pharmacological activities including antifungal effects [5,9]. However, the specific pathways involved in the growth and development of *S. rugosoannulata* and the synthesis of its bioactive components remain unclear, which has severely hindered related research on gene function and breeding of *S. rugosoannulata*.

To systematically investigate the functions of genes involved in the growth and development of fruiting bodies and the biosynthesis of secondary metabolites in *S. rugosoannulata*, a range of molecular genetic tools, such as genetic transformation, target-gene functional analysis, selectable markers, and reporter genes, is essential. To date, multiple DNA-genetic transformation methods have been successfully established in fungi, including protoplast-mediated transformation (PMT) [10], electroporation transformation [11], Agrobacterium-mediated transformation (AMT) [12], biolistic transformation [13], and shock-wave-mediated transformation [14]. The *Agrobacterium-tumefaciens*-mediated transformation (ATMT) has been extensively employed in commercially valuable edible and/or medicinal fungi, including *Agrocybe aegerita* [15], *Ophiocordyceps sinensis* [16], *Flammulina velutipes* [17], and *Tremella fuciformis* [18], owing to its high transformation efficiency, minimal equipment requirements, and low-copy integration of target genes. Nevertheless, the genetic transformation system for *S. rugosoannulata* remains underdeveloped, with no experimental evidence currently available to substantiate the in vivo expression of either homologous or heterologous genes in *S. rugosoannulata*.

The rational selection of screening marker genes and reporter genes is crucial for establishing a stable genetic transformation system. To date, numerous studies have provided a wide range of screening marker genes and reporter genes. Common screening markers can be categorized into nutritional deficiency type markers (such as *argB* [19], *pyrG* [20], *trpC* [21], and *ura5* [22]) and resistance markers (*hph* [23], *bar* [24], *nat1* [25], and *ble* [26]). However, the use of auxotrophic markers requires obtaining auxotrophic strains first, which complicates the experimental process. Moreover, studies have shown that exogenous resistance markers often exhibit low expression levels, are prone to loss, and pose biosafety concerns. Therefore, developing stable and reliable homologous resistance genes as selection markers for target strains is of paramount importance. Reporter genes play a significant role in assessing promoter strength, gene expression patterns, and protein localization. Commonly used reporter genes in fungal genetic transformation include *GUS* [12,27] and fluorescent protein genes, such as *DsRed*, *eGFP*, and *mCherry* [28,29,30].

In this study, we successfully established a stable genetic transformation system for *S. rugosoannulata* protoplasts mediated by *Agrobacterium tumefaciens* EHA105. Additionally, the endogenous promoter of glyceraldehyde-3-phosphate dehydrogenase (GPD) and the iron-sulfur protein subunit of succinate dehydrogenase (SDI) in *S. rugosoannulata* were identified and designated as P*_SrGPD_* and P*_SrSDI_*, respectively. These promoters were used to drive the expression of target genes in *S. rugosoannulata*. Through ATMT systems, we endowed *S. rugosoannulata* with two resistance selection markers (the endogenous mutated *SDI* gene and the exogenous *hph* gene) and achieved the expression of two exogenous reporter genes (*mCherry* and *GUS*). The development of the *S. rugosoannulata* ATMT systems and the expression of marker genes and reporter genes provide genetic tools for subsequent research on the functions of other genes and precision breeding in *S. rugosoannulata*.

## 2. Results

### 2.1. Characterization of the Wild-Type GPD and SDI Gene from S. rugosoannulata

**Figure 1:**
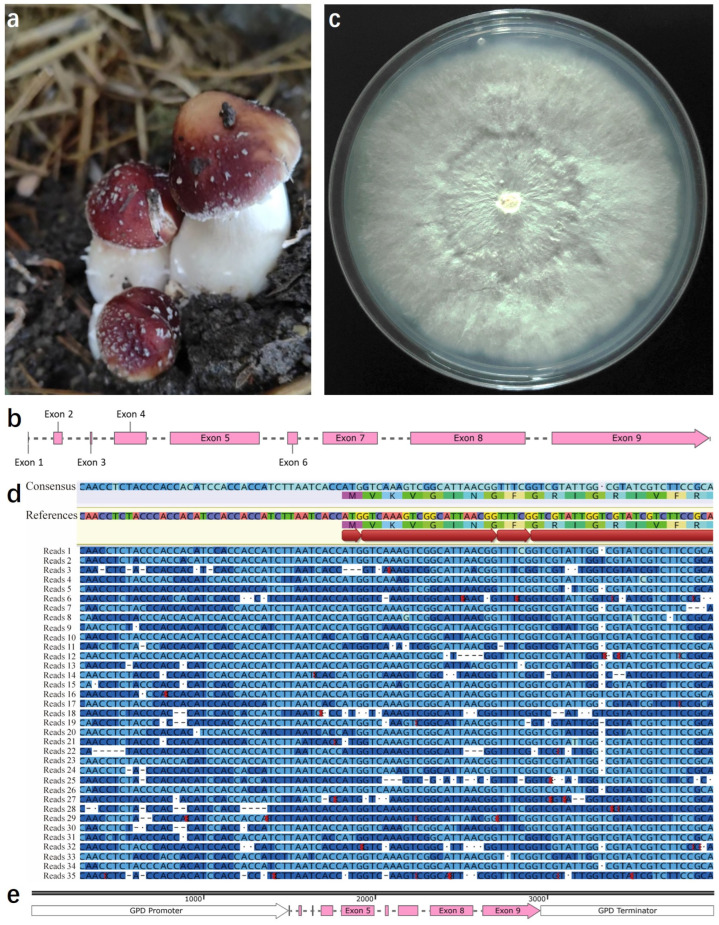
Characterization of the *gpd* in *S. rugosoannulata*. (**a**) mature fruiting bodies of *S. rugosoannulata*; (**b**) linear model of the predicted coding region for *SrGPD*; (**c**) morphology of *S. rugosoannulata* mycelium grown on modified potato dextrose agar (mPDA) medium; (**d**) alignment of transcripts obtained via full-length transcriptome sequencing against the reference sequence of *SrGPD*; (**e**) comprehensive structural model of *SrGPD*, including the promoter, coding region, and terminator.

In fungi, the *GPD* gene exhibits high expression levels, and its promoter has been selected to drive the expression of target genes [31,32]. Following the annotation analysis of the *S. rugosoannulata* genome (Figure 1a), the gene encoding the GPD protein was predicted to be *SrGPD*. The predicted coding sequence (CDS) of *SrGPD* is 1017 bp long and spans nine exons (Figure 1b). To validate the accuracy of the *SrGPD* gene prediction, the total RNA from the mycelium of *S. rugosoannulata* (Figure 1c) was extracted, PCR was performed using cDNA synthesized from mRNA of *S. rugosoannulata* with specific primers, and the PCR products were sequenced (Figure S1). Additionally, full-length transcriptome sequencing was conducted on *S. rugosoannulata* mRNA, and the alignment results of *SrGPD* against the reference sequence are shown in Figure 1d. At the amino acid level, GPD shares 85.92% identity with SrGPD and clusters closely with other fungal GPDs, indicating a high level of evolutionary conservation (Figure S2). SrGPD is a member of the PLN02272 superfamily, and its sequence alignment results are presented in Figure S3. Phylogenetic analysis further confirmed the orthology of SrGPD (Figure S4, Table S4). By integrating these findings, the 1500 bp non-coding region upstream of the *SrGPD* start codon was defined as the promoter region, the potential promoter elements that may exist were predicted (Figures S5 and S6), and the 1000 bp non-coding region downstream of the stop codon was defined as the terminator region (Figure S7). Consequently, the complete gene structure of *SrGPD*, as depicted in Figure 1e, includes a coding region of 1464 bp with eight introns, confirming the predicted CDS length.

**Figure 2:**
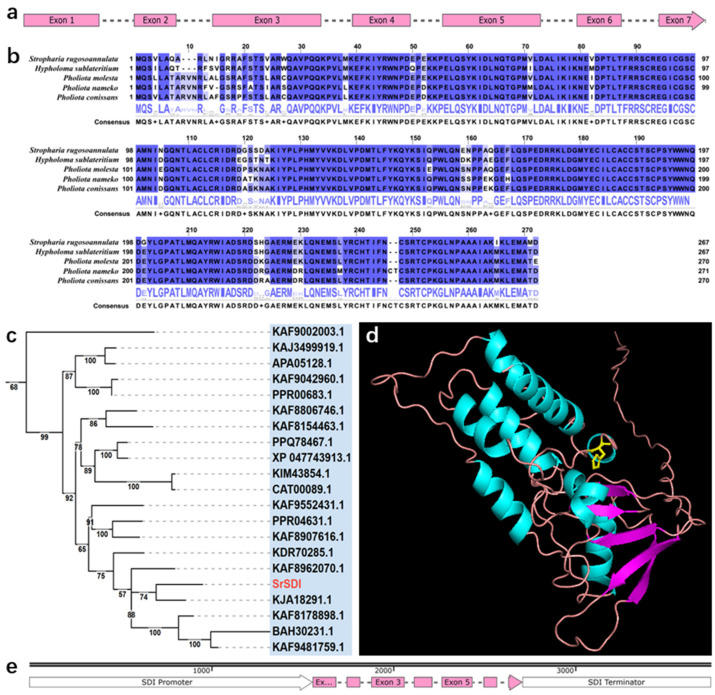
Characterization of the *SDI* in *S. rugosoannulata*. (**a**) linear model of the predicted coding region for the *SrSDI* gene; (**b**) multiple sequence alignment results analyzing the conservation differences between the *SrSDI* protein and those from other species; (**c**) partial phylogenetic tree based on the *SrSDI* protein sequence, with all species shown belonging to the Agaricineae suborder; (**d**) schematic diagram of the three-dimensional structure of the *SrSDI* protein, obtained via homology modeling using Swiss-Model; (**e**) comprehensive structural model of *SrSDI*, including the promoter, coding region, and terminator.

Similarly, the *SrSDI* gene in *S. rugosoannulata* was characterized. By integrating gene prediction, full-length transcriptome sequencing, and Sanger sequencing results, the coding region of *SrSDI* was found to be 1151 bp long, comprising 7 exons and 6 introns (Figure S8), with a coding sequence length of 804 bp. The gene structure is depicted in Figure 2a. Additionally, the full-length transcriptome alignment results of *SrSDI* against the reference sequence are shown in Figure S9. To identify conserved amino acid positions conferring carboxin resistance in *SrSDI*, the *SrSDI* amino acid sequence was aligned with those from various fungal species (Figure 2b). A phylogenetic tree of the *SDI* gene across different fungi was also constructed (Figure 2c and Figure S10, Table S5). The *SrSDI* is a member of the PLN00129 superfamily, and its sequence alignment results are presented in Figure S10. Based on previous reports in the literature [15,33], the histidine codon CAT at position 238 of the *SrSDI* amino acid sequence was replaced with the leucine codon CTT to generate the putative carboxin-resistant allele *SrSDI*^R^ (Figure 2d and Figure S11). Subsequently, *SrSDI*^R^ was ligated with the native *SrSDI* promoter (Figure S12) and terminator (Figure S13) and inserted into a transformation vector to serve as an element conferring carboxin resistance to *S. rugosoannulata* (Figure 2e).

### 2.2. The Monokaryon Mycelium of S. rugosoannulata Exhibits Sensitivity to Hygromycin B and Carboxin

**Figure 3:**
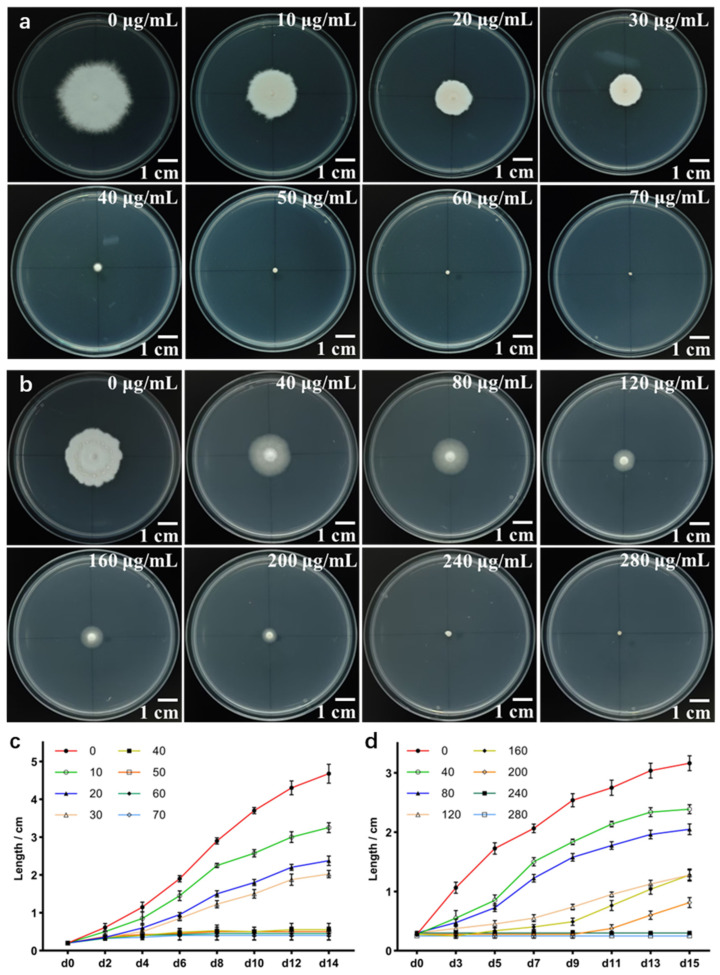
Effects of different concentrations of hygromycin B and carboxin on mycelial growth inhibition of *S. rugosoannulata*. (**a**) growth status of *S. rugosoannulata* mycelia on mPDA plates containing varying concentrations of hygromycin B; (**b**) growth status of *S. rugosoannulata* mycelia on mPDA plates containing varying concentrations of carboxin; (**c**) changes in mycelial diameter under different concentrations of hygromycin B treatment; (**d**) changes in mycelial diameter under different concentrations of validamycin treatment. The *x*-axis indicates the number of days of mycelial growth on the medium, while the *y*-axis shows the elongation of mycelial diameter measured using the cross-measurement method in (**c**,**d**) (three replicates were set for each group).

The hygromycin resistance gene, initially isolated from *Escherichia coli*, has been widely applied in genetic transformation studies of plants and various fungi [34,35]. In this study, the monokaryon mycelium of *S. rugosoannulata* (HC7) was inoculated on mPDA medium supplemented with different concentrations of hygromycin B. It was found that a concentration of 50 μg/mL hygromycin B significantly inhibited the growth of HC7 mycelia on the modified PDA plates (Figure 3a,c).

Mutations at key sites such as C_S42 or B_H216 of the *SDI* gene in fungi decreases the strength of the water-bridged hydrogen-bond network between the mitochondrial succinate-ubiquinone oxidoreductase (SQR) and the NH group of the amide bond of carboxin. This significantly reduces the binding affinity of carboxin for the SQR target, thereby conferring resistance to the fungicide in fungi. In contrast, the wild-type enzyme maintains an intact hydrogen-bonding network that allows for strong and effective binding of carboxin, resulting in no resistance [36]. Generally, heterologous genes containing non-native components exhibit relatively low expression efficiency in basidiomycetes. For example, the hygromycin resistance gene, which originates from Escherichia coli, often shows limited stability and expression efficiency in fungi. In contrast, the endogenous mutated SDI gene demonstrates higher stability and stronger expression efficiency, making it a widely applied marker in fungal genetic transformation studies [37,38]. The experimental results showed that when the concentration of carboxin was 240 μg/mL, the growth of HC7 mycelia was completely inhibited (Figure 3b,d). These findings indicate that both hygromycin B and carboxin can serve as effective selection agents for transformants in *S. rugosoannulata* genetic transformation experiments.

### 2.3. Establishment of a Transformation Protocol for HC7

**Figure 4:**
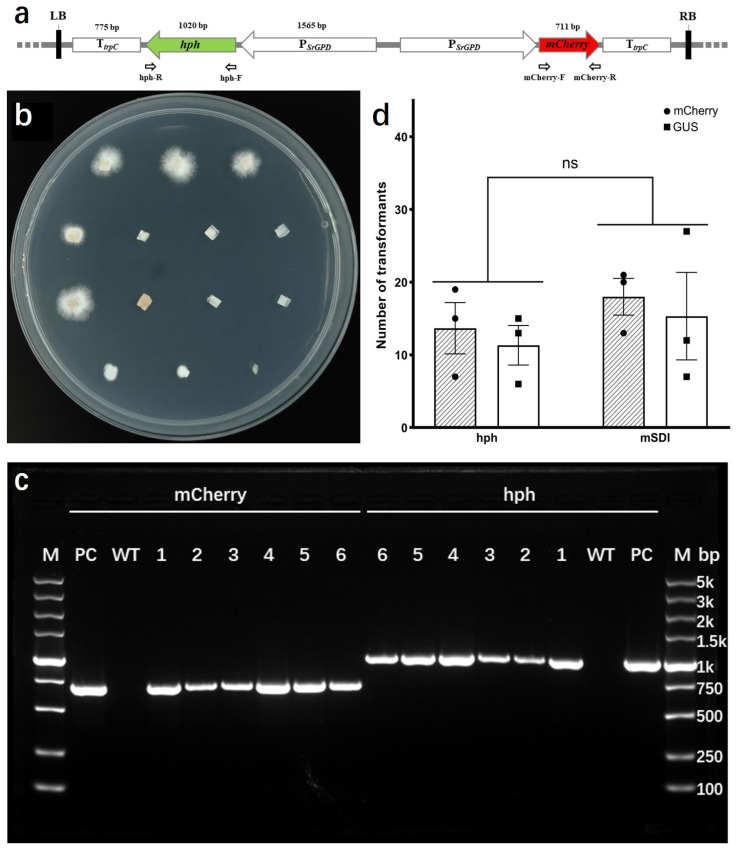
Protoplast transformation of HC7 and identification of transformants. (**a**) Schematic representation of the structure of plasmid pCM-GHT-GMT. Primers hph-F and hph-R were used to verify the presence of the hph in the transformants, while primers mCherry-F and mCherry-R were used to confirm the presence of the mCherry in the transformants. (**b**) Growth status of *S. rugosoannulata* transformants using plasmid pCM-GHT-GMT on re-screening plates. (**c**) Identification of insertion fragments from plasmid pCM-GHT-GMT in *S. rugosoannulata* transformants using the primer pairs described in panel (**a**). PC, plasmid positive control; WT, wild type; 1–6, transformants after re-screening; M, DNA molecular marker. (**d**) Transformation efficiency of *S. rugosoannulata* protoplasts using different plasmids. ns, no significant.

Resistant transformants were selected using regeneration medium supplemented with 50 μg/mL hygromycin B for HC7 protoplasts transformed with pCM-GHT-GMT and pCM-GHT-GGT or 240 μg/mL carboxin for HC7 protoplasts transformed with pCM-mSDI-GMT and pCM-mSDI-GGT (Figure 4a and Figure S14). Colonies grown on the regeneration medium (RM) were transferred to mPDA plates containing either 50 μg/mL hygromycin B or 240 μg/mL carboxin for re-screening (Figure 4b and Figure S15). Vigorously growing transformants from the re-screening plates were individually expanded, and their genomic DNA was extracted. PCR amplification of T-DNA fragments (such as *hph*, *mCherry*, *GUS*, and *mSDI*) confirmed the presence of the transferred sequences (Figure 4c and Figure S16). Additionally, PCR was used to detect potential contamination by *A. tumefaciens* DNA outside the T-DNA borders in the genomic samples, as shown in Figure S17. Statistical analysis revealed that the regeneration efficiency of HC7 protoplasts was measured to be 1.07% (Figure S18). Among these, the transformation efficiency of *S. rugosoannulata* protoplasts mediated by the resistance gene of *hph* was relatively low, yielding approximately 13 transformants per 10^6^ protoplasts, with a transformation efficiency of about 1.17‰. In contrast, in transformations mediated by *mSDI* as the resistance gene, approximately 17 transformants per 10^6^ protoplasts were obtained, resulting in a transformation efficiency of about 1.56‰ (Figure 4d).

### 2.4. Identification of the Inserted T-DNA Flanking Sequences in Transformants

**Figure 5:**
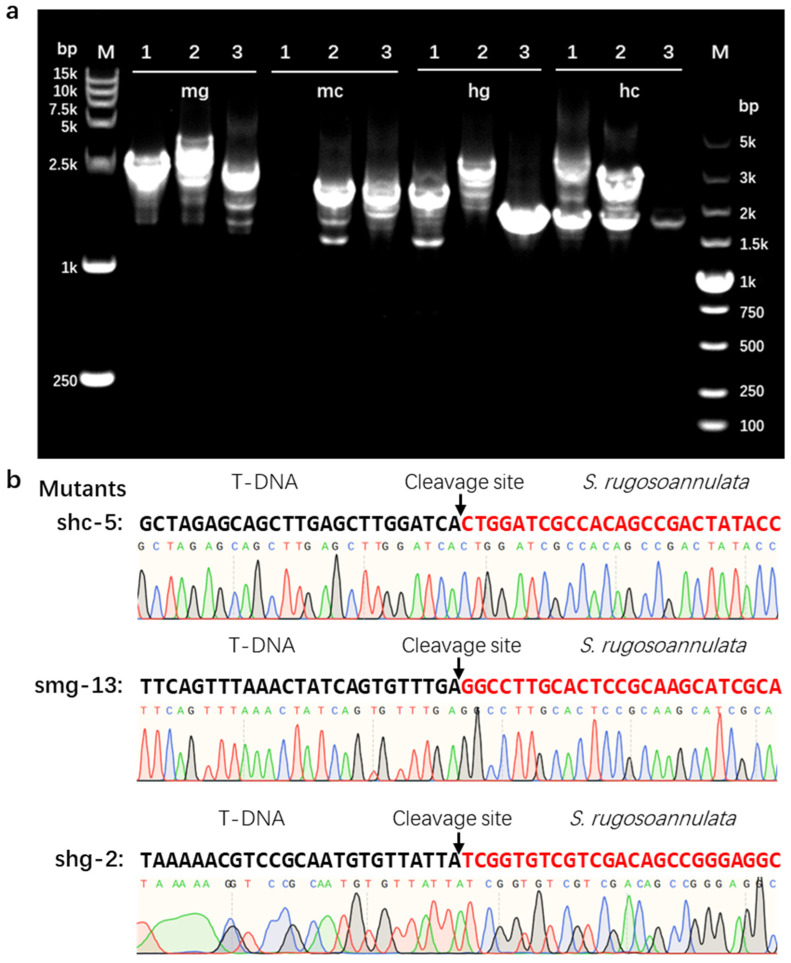
Identification of flanking sequences in transformants. (**a**) Agarose gel electrophoresis results of mhiTAIL-PCR products. Three positive transformants were selected from each of the four plasmid-transformed lines for mhiTAIL-PCR analysis. Specifically, mg, mc, hg, and hc refers to transformants harboring T-DNA from plasmid pCM-mSDI-GGT, pCM-mSDI-GMT, pCM-GHT-GGT, and pCM-GHT-GMT, respectively. M, DNA molecular weight marker. (**b**) Schematic representation of the sequence at which T-DNA is joined to its flanking regions. The base sequences in black capital letters originate from the recombinant plasmid DNA fragments, while those in red capital letters represent genomic DNA sequences of *S. rugosoannulata*. The position indicated by the black arrow denotes the insertion site of T-DNA within the *S. rugosoannulata* genome.

The flanking sequences of the T-DNA inserted in the positive transformants were identified using mhiTAIL-PCR. The amplified products ranged from 0.5 kb to 3 kb (Figure 5a). The T-DNA flanking sequences of the transformants were aligned against the *S. rugosoannulata* reference genome to confirm the insertion sites of T-DNA in the HC7 genome (Figure 5b). Among the selected positive transformants, the right end of the T-DNA inserted into shc-5 was truncated 39 bases away from the RB border sequence; the left end of the T-DNA inserted into shg-2 was truncated 20 bases away from the LB border sequence; and the truncation position of the right end of the T-DNA inserted into smg-13 was located on RB. Sequence analysis near the T-DNA insertion sites revealed that the insertion site in transformant sch-5 corresponded to the predicted gene S003.1G004910.1, which encodes a natural resistance-associated macrophage protein. In contrast, the insertion sites in transformants shg-2 and smg-13 were not located within predicted genes.

### 2.5. Detection and Visualization of mCherry

**Figure 6:**
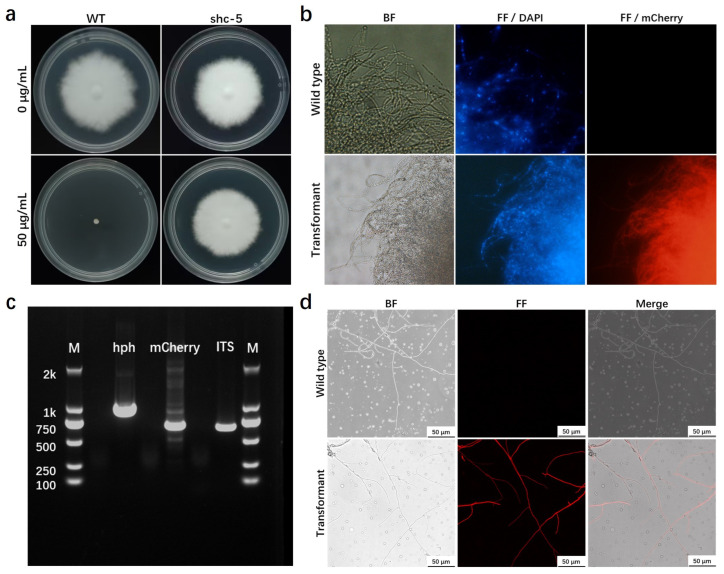
Expression and fluorescence detection of mCherry in *S. rugosoannulata*. (**a**) Growth comparison of wild-type (upper images) and mCherry-expressing transformant shc-5 (lower images) on mPDA medium with (left images) or without (right images) hygromycin B. (**b**) Fluorescence imaging analysis of mycelia from wild-type and transformant shc-5. The images display the mycelia of wild-type (upper images) and transformant (lower images) under bright-field and fluorescence modes. BF, bright field; FF, fluorescence mode, showing DAPI staining and mCherry expression. (**c**) Agarose gel electrophoresis analysis of transformants. PCR amplification of genomic DNA from positive transformant shc-5 using primer pairs hph-F/hph-R (lane 1), mCherry-F/mCherry-R (lane 2), and ITS1/ITS4 (lane 3). M, DNA molecular marker. (**d**) Laser confocal microscopy imaging analysis of mycelia from wild-type and transformant shc-5. The images depict the mycelia of wild-type (upper images) and transformant (lower images) under bright-field and fluorescence modes, as well as the merged images.

The fluorescence microscopy was used to observe multiple-passage positive transformants (Figure 6a) generated by ATMT with the recombinant plasmid pCM-GHT-GMT to detect mCherry expression. The results are shown in Figure 6b. PCR analysis was conducted on the transformants exhibiting stronger fluorescence obtained from the pCM-GHT-GMT-mediated transformation (Figure 6c). Subsequently, laser confocal microscopy was used to further examine the distribution of fluorescence within hyphae, as shown in Figure 6d. No fluorescence was detected in the control group, while the experimental group showed uniform distribution of mCherry fluorescence signals within the hyphae, indicating stable expression of the mCherry protein in the transformants.

### 2.6. Detection of GUS Gene Expression

**Figure 7:**
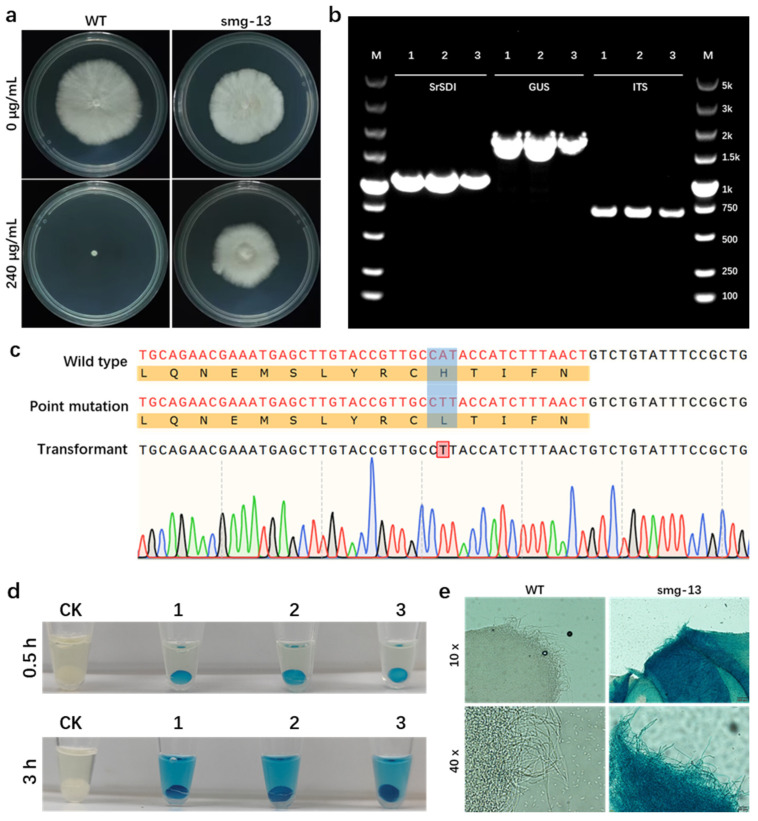
Expression and fluorescence detection of GUS in *S. rugosoannulata*. (**a**) Growth comparison of wild-type (left images) and mCherry-expressing transformant smg-13 (right images) on mPDA medium with (upper images) or without (lower images) carboxin. (**b**) Agarose gel electrophoresis analysis of transformants. PCR amplification of genomic DNA from positive transformant smg-13 using primer pairs SDI-F/SDI-R (lane 2–4), GUS-F/GUS-R (lane 5–7), and ITS1/ITS4 (lane 8–10). M, DNA molecular marker. (**c**) Sanger sequencing results of transformants obtained using plasmid pCM-mSDI-GGT. The 238th histidine (CAT) in the wild-type *SrSDI* was mutated to leucine (CTT), highlighted with blue background, to confer carboxin resistance to the transformants, and this mutation was detected in the transformants (bases highlighted with red background). (**d**) GUS staining results of different transformants. Tubes 1–3 display the GUS staining situation for three different transformants. CK, wild type. (**e**) Microscopic images of hyphae from positive transformants. The images depict the hyphae of the wild type (left) and the transformant smg-13 (right) before and after GUS staining.

The positive transformants generated by ATMT with the recombinant plasmid pCM-mSDI-GGT (Figure 7a) were selected. After PCR verification and sequencing (Figure 7b,c), these transformants were stained using the X-gluc staining solution, and the results are presented in Figure 7d. The blue precipitates were observed in the EP tubes containing the mycelia of positive transformants, indicating successful expression and functional activity of the GUS, which catalyzed the conversion of X-gluc into blue precipitates. After decolorization, the mycelia were examined under an inverted microscope (Figure 7e). The mycelia of the transformants were uniformly stained blue, while the untransformed mycelia remained colorless, further confirming the catalytic activity of GUS in the transformants.

## 3. Discussion

This study demonstrated that both the exogenous hygromycin resistance gene and the homologous mutant *SDI* gene can serve as effective genetic transformation screening markers for *S. rugosoannulata*. By making appropriate modifications to the ATMT system originally developed for *Morchella importuna* [39] and optimizing the protoplast preparation method for *S. rugosoannulata*, we successfully established an ATMT system suitable for genetic transformation of *S. rugosoannulata* HC7 protoplasts. After co-cultivating the induced *A. tumefaciens* EHA105 with HC7 protoplasts for 36 h, the mixture was transferred to regeneration medium plates containing the corresponding antibiotics. Through multiple rounds of selection, a significant number of transformants were obtained. These results indicate the feasibility and effectiveness of the ATMT system in *S. rugosoannulata*.

Overall, fungal ATMT transformation is influenced by multiple factors, including *Agrobacterium* strain, fungal recipient, co-culture conditions, and selection markers [40]. To achieve optimal transformation efficiency and obtain the maximum number of transformants, it is essential to optimize these influencing factors [41,42,43]. The optimal temperature for *S. rugosoannulata* mycelial growth is generally 24–27 °C, with an optimal pH range of 5–7 [44]. Transformation efficiencies mediated by different *A. tumefaciens* strains can vary significantly. For example, LBA4404 can produce hygromycin-resistant transformants in *Flammulina velutipes*, whereas no transformants were obtained using AGL-1 [45]. In *Pleurotus ostreatus*, *Agrobacterium* strains GV3101, EHA105, and LBA4404 exhibit varying transformation efficiencies [46]. Our studies have shown that co-culturing HC7 protoplasts with EHA105 at 25 °C and pH 5.5 for 36 h yields a substantial number of transformants.

The T-DNA flanking sequences were analyzed using the mhiTAIL-PCR technique. It was inferred that the genes potentially affected by the insertion site, such as S003.1G004910.1, encode a natural resistance-associated macrophage protein. Disruption of the functional sequences of these related genes may influence the morphology, growth and development, and stress tolerance of the transformants. In the absence of selection pressure, resistance gene loss was observed in 11% of positive transformants. This phenomenon has been reported in various fungi, including *Mucor miehei* [47], *Aspergillus sojae* [48], and *Ophiocordyceps sinensis* [16]. Research suggests that transposons present in the genome may contribute to reduced T-DNA stability [49,50,51]. It is hypothesized that T-DNA loss in *S. rugosoannulata* may be associated with specific characteristics of its genome.

As a straw-rotting fungus, *S. rugosoannulata* is typically cultivated in agricultural settings using rice straw, wheat straw, and other crop residues as substrates, with a biological conversion efficiency of approximately 45%. For optimal harvest, *S. rugosoannulata* should be collected when the mycelial membrane has not ruptured and the pileus remains curled and unexpanded. However, under unfavorable environmental conditions, such as high temperatures, *S. rugosoannulata* tends to prematurely expansion its pileus. The low biological conversion efficiency and the tendency to open prematurely under unsuitable conditions pose significant challenges for agricultural cultivation. Current research indicates that genes such as *exg2* [52] and *exp1* [53] play crucial roles in the pileus expansion of basidiomycetes, suggesting that the pileus expansion mechanism of *S. rugosoannulata* may also be regulated by similar genes. This hypothesis should be verified through genomic analysis. Different strains of *S. rugosoannulata* exhibit varying abilities to degrade substrate such as straw [54,55], and the underlying mechanisms require further investigation. Through this ATMT system, studies on the deletion, overexpression, or silencing of genes related to cellulose degradation and fruiting body development can provide valuable insights into improving agricultural practices. Such research can ultimately enhance the economic benefits of *S. rugosoannulata* cultivation by optimizing both yield and quality.

The ATMT system developed in this study facilitates more in-depth molecular genetics research on *S. rugosoannulata*. By fusing functional protein genes with reporter genes such as *mCherry*, the localization of gene expression products of interest can be precisely studied [56,57]. In a recent study on *Arthrobotrys flagrans*, the *GprC* gene was fused to a C-terminal green fluorescent protein (GFP) reporter, and its distribution in the apical and subapical regions of *A. flagrans* hyphae was observed using fluorescence microscopy, enabling the subcellular localization of the GprC protein [56]. Furthermore, by replacing components on plasmids like pCM-mSDI-GMT constructed in this study to build new recombinant vectors and utilizing the ATMT system for transformation, overexpression or silencing of target genes becomes more feasible, similar to approaches used in *Grifola frondosa* [58] and *Cordyceps militaris* [59]. Additionally, CRISPR/Cas9 technology has advanced precise genome editing in many fungi, including gene insertion, deletion, base conversion, and transcriptional activation [12,60,61]. In particular, Kamiya et al. demonstrated that transient expression of a CRISPR/Cas9 cassette enabled efficient homologous recombination in *Lentinula edodes*, overcoming the low natural recombination efficiency in this species [61]. Integrating CRISPR/Cas9 with the ATMT system will enable precise genome editing, gene knockout, and pathway regulation studies in *S. rugosoannulata*.

## 4. Conclusions

In this research, a series of recombinant vectors were constructed, in which the Hph^R^ cassette (composed of P*_SrGPD_*, *hph*, and T*_AnTrpC_*) or Cbx^R^ cassette (composed of P*_SrSDI_*, mutSDI, and T*_SrSDI_*) served as resistance screening markers, and *mCherry* or *GUS* acted as a reporter gene. Based on the aforementioned plasmids, the ATMT-based *S. rugosoannulata* HC7 protoplast transformation method was established, and the aforementioned screening markers and reporter genes were successfully functionalized. The achievement of the *S. rugosoannulata* protoplast ATMT system makes it possible to investigate the functions of target genes of interest in *S. rugosoannulata* and provides an essential genetic tool for subsequent studies on the molecular mechanism and gene function of *S. rugosoannulata*.

## 5. Materials and Methods

### 5.1. Strains and Culture Conditions

The *S. rugosoannulata* monokaryon strain (HC7) was obtained from our laboratory’s previous screening and utilized in all experiments of this study. The purified mycelium was maintained on mPDA composed of 8.0 g/L potato extract powder, 20.0 g/L dextrose, 0.5 g/L MgSO_4_·7H_2_O, 1.0 g/L KH_2_PO_4_, 2.0 g/L peptone, and 18.0 g/L agar at 25 °C. The *Escherichia coli* DH5α, used for plasmid transformation, amplification, and preservation, was cultured on Luria-Bertani (LB) medium supplemented with ampicillin or kanamycin at 37 °C. *A. tumefaciens* EHA105, which serves as the donor of T-DNA, was employed for fungal transformation. The induction medium (IM) [62] was used to induce *A. tumefaciens* virulence factors. Co-cultivation of EHA105 and *S. rugosoannulata* protoplasts was conducted using the co-culture medium (CIM) [62]. For regeneration and selection of transformed protoplasts, the RM composed of 8.0 g/L potato extract powder, 20.0 g/L sucrose, 1.5 g/L MgSO_4_·7H_2_O, 1.5 g/L KH_2_PO_4_, 1.5 g/L K_2_HPO_4_·3H_2_O, 2.0 g/L yeast extract, 2.0 g/L peptone, 0.1 g/L vitamin B_1_, 0.1 g/L vitamin B_6_, 109.3 g/L mannitol, and 15.0 g/L agar containing 200 μg/mL cefotaxime sodium salt (Solarbio, Beijing, China) was supplemented with either 50 μg/mL hygromycin B or 240 μg/mL carboxin.

### 5.2. Identification of Candidate Genes and Their Regulatory Sequences

To identify the candidate genes encoding GPD and SDI in HC7, we retrieved the published *S. rugosoannulata* genome sequence from the NCBI database, as well as *GPD* and/or *SDI* gene sequences from closely related species, such as *Flammulina velutipes*, *Pleurotus ostreatus*, *Hypsizygus marmoreus*, and *Lentinula edodes*. BLASTP was employed to compare these sequences against the genome of *S. rugosoannulata* to obtain homologous sequences for the gene of *GPD* and *SDI*. Multiple sequence alignment was performed using Muscle, and phylogenetic analysis was conducted using IQ-TREE. To avoid potential errors in genome annotation, the full-length RNA sequencing of HC7 mycelia was carried out using Oxford Nanopore Technologies (ONT). Primers were designed based on the reference genome sequence, and gene annotation results of *S. rugosoannulata*, and PCR amplification followed by Sanger sequencing was used to confirm the gene sequences of *GPD* and *SDI* in HC7.

### 5.3. Extraction of Genomic DNA and Long-Read RNA-Seq

Genomic DNA was extracted from the mycelium by employing the Fungal Genomic DNA Extraction Kit (Sangon Biotech, Beijing, China). Total RNA was isolated from the mycelium with TRIzol^TM^ Reagent (Thermo Fisher Scientific, Waltham, MA, USA), and cDNA was synthesized using PrimeScript^TM^ IV 1st strand cDNA Synthesis Mix (TaKaRa, Kyoto, Japan). The Oxford Nanopore long-read RNA-Seq was conducted on the PromethION platform at Biomarker Technology Company (Beijing, China).

### 5.4. Construction of Plasmid

The plasmid pMD19-T (Takara, Kyoto, Japan) was used for the preservation of plasmid elements, while pCAMBIA1300 served as the backbone vector for genetic transformation. The *hph*, *mCherry*, and *GUS* genes were obtained from the laboratory-preserved plasmids pGH-*hph*, pGH-*mCherry*, and pGH-*GUS*, respectively. The *TrpC* terminator (T*_AnTrpC_*) sequence was amplified from plasmid pAN7-1. The sequences of *GPD* promoter, including the first exon, the first intron, and the initial 9 base pairs of the second exon (P*_SrGPD_*), *SDI* gene encoding SDI, *SDI* gene promoter (P*_SrSDI_*), and *SDI* gene terminator (T*_SrSDI_*), were all amplified from the HC7 genome.

The primer pairs hph-F/hph-R, mCherry-F/mCherry-R, GUS-F/GUS-R, and TtrpC-F/TtrpC-R were used to amplify the *hph*, *mCherry*, *GUS*, and T*_AnTrpC_* sequences from plasmids pGH-*hph*, pGH-*mCherry*, pGH-*GUS*, and pAN7-1, respectively. The GPDi-F/GPDi-R primer pair was used to amplify the P*_SrGPD_* promoter from the HC7 genome. The SDI-F/SDI-R primer pair was used to amplify the *SDI* cassette, including the promoter (P*_SrSDI_*) and terminator (T*_SrSDI_*) of the *SDI* gene, from the HC7 genome, and the amplified product was designated as *oriSDI*. To generate a mutant version of *SDI* (*mutSDI*), primers SDI-F/mut-SDI-R and mut-SDI-F/SDI-R were used to amplify *oriSDI*, followed by gene splicing by overlap extension PCR (SOE PCR). Both the *oriSDI* (*oSDI*) and *mutSDI* (*mSDI*) sequences were ligated into the pMD18-T vector, resulting in recombinant vectors pMD-*oSDI* and pMD-*mSDI*, respectively (Figure S19). The pCM-F/pCM-R and SP-F/SP-R primer pairs were used to amplify the backbone sequence (pCM) and spacer sequence (SP) from the pCAMBIA1300 plasmid. Homologous arms were added to the hph, mCherry, and GUS fragments using the primer pairs homo-GH/homo-HT, homo-GM/homo-MT, and homo-GG/homo-GT, respectively. The SOE PCR was then performed to connect the P*_SrGPD_* promoter, the homologous-arm-modified hph, mCherry, and GUS fragments, and the T*_AnTrpC_* terminator. The resulting constructs were cloned into the pMD18-T vector via TA cloning, yielding the recombinant vectors pMD-GHT, pMD-GMT, and pMD-GGT (Figure S19).

To construct the binary vector pCM-GHT-GMT, PCR amplification was performed on the recombinant vectors pMD-GHT and pMD-GMT using primers homo-GTS/homo-BGT and homo-SGT/homo-GTB, respectively. The amplified fragments were then ligated with the backbone sequence pCM and the spacer sequence SP via homologous recombination. Similarly, the binary vector pCM-GHT-GGT was constructed using the same primer pairs for PCR amplification of pMD-GHT and pMD-GGT. For the construction of the binary vector pCM-mSDI-GMT, PCR amplification was conducted on the recombinant vectors pMD-mSDI and pMD-GMT using primers homo-BMS/homo-MSS and homo-SGT/homo-GTB, respectively. The resulting fragments were ligated with the backbone sequence pCM via homologous recombination. Likewise, the binary vector pCM-mSDI-GGT was obtained by performing PCR amplification on pMD-mSDI and pMD-GGT using the same primer pairs and subsequent ligation with pCM via homologous recombination. The primers used for PCR amplification and plasmid construction are listed in Table S1.

### 5.5. Test of Fungal Sensitivity Toward Hygromycin B and Carboxin

The mycelial blocks of HC7 were inoculated onto mPDA medium supplemented with different concentrations of hygromycin (0, 10, 20, 30, 40, 50, 60, and 70 μg/mL) or carboxin (0, 40, 80, 120, 160, 200, 240, and 280 μg/mL). Cultures were incubated in the dark at 25 °C for 2 weeks. Mycelial growth was recorded to evaluate the sensitivity of HC7 to hygromycin and validamycin.

### 5.6. Protoplast Preparation

The method for preparing protoplasts of *S. rugosoannulata* was adapted primarily from the protocol described in previous studies [63,64], with several modifications. Specifically, mycelial blocks of HC7 were harvested from mPDA plates using a 0.5 cm diameter circular punch. These blocks were then homogenized and transferred to 250 mL of liquid mPDA medium (without agar) and incubated in darkness at 25 °C with shaking at 180 rpm for 6 days. Following this, the mixture was centrifuged at 3000× *g* for 10 min, after which the supernatant was discarded. The mycelia were subsequently washed twice with 0.6 M sorbitol solution, discarding the supernatant each time. For every 200 mg of mycelia, 1 mL of freshly prepared 1.5% (*m*/*v*) lywallzyme (Guangdong microbial culture collection center) solution was added, and the suspension was incubated in darkness at 25 °C for 3 h. The resulting mixture was filtered through a 10 μm cell strainer, and the filtrate was collected in a 50 mL centrifuge tube and centrifuged again at 3000× *g* for 10 min. The protoplast pellet was then gathered, washed twice with 0.6 M mannitol, and resuspended in an appropriate volume of 0.6 M mannitol to yield purified protoplasts.

### 5.7. Agrobacterium-tumefaciens-Mediated Transformation of S. rugosoannulata

The *A. tumefaciens* EHA105 harboring different plasmids was inoculated into LB medium supplemented with 20 μg/mL rifampicin and 50 μg/mL kanamycin and cultured at 28 °C with shaking at 200 rpm until the OD_600_ value reached 0.6 to 0.8. After centrifugation, the bacterial pellet was resuspended in IM medium containing 200 μM acetosyringone (AS) and adjusted to an OD_600_ of approximately 0.2. The culture was then incubated under dark conditions at 28 °C with shaking at 200 rpm until the OD_600_ value reached 0.6 to 0.8. The induced EHA105 suspension was mixed in equal volume with *S. rugosoannulata* protoplasts and incubated statically at 25 °C for 30 min before being transferred onto a CIM plate (containing 200 μM AS) covered with nitrocellulose membrane. Co-cultivation was performed in the dark at 25 °C for 48 h. Subsequently, the nitrocellulose membrane was transferred to an RM plate containing appropriate antibiotics and cultured at 25 °C until transformants emerged. All experiments were conducted in triplicate.

### 5.8. PCR Analysis of Putative Transformants

The transformants that grew on the regeneration medium were transferred to mPDA medium supplemented with the corresponding antibiotics (50 μg/mL hygromycin B or 240 μg/mL carboxin) for secondary screening and incubated in the dark at 25 °C for 7 days. Genomic DNA was extracted from the transformants, and PCR identification of the transformants obtained by ATMT was performed using specific primers (Table S1) and ITS primers (Table S2). The amplification products were analyzed by agarose gel electrophoresis and Sanger sequencing. Positive transformants were confirmed based on the position of the amplification product bands and the Sanger sequencing results. PCR was performed on the transformed genome using the primers listed in Table S2 to exclude potential contamination from bacterial DNA. Furthermore, mhiTAIL-PCR [65] was employed to clone the flanking sequences of the left or right T-DNA borders from the selected transformants. The primers used for mhiTAIL-PCR are presented in Table S3. The resulting T-DNA flanking sequences were aligned against the reference genome of *S. rugosoannulata* to verify the precise insertion site and assess whether the functional genes of HC7 were disrupted.

### 5.9. Fluorescence Observation and GUS Activity Analysis

To observe mCherry expression in transformants, the mycelia were cultivated on mPDA medium without antibiotics and cultured for 7 days. The mycelia were placed on a glass slide for preliminary examination under an inverted fluorescence microscope (Olympus, IX73P2F, Tokyo, Japan) at 400x magnification. Hyphae exhibiting mCherry fluorescence were selected and examined using a laser confocal microscope (Leica, Stellaris 5, Wetzlar, Germany) with excitation at 587 nm and peak emission at 610 nm, corresponding to its characteristic fluorescence peak. Images were acquired using a 400× objective lens. To assess the activity of GUS in the positive transformants, mycelia were scraped from fresh plates and resuspended in the GUS staining solution (0.5 mg/mL X-Gluc, 100 mM phosphate buffer [pH 7.0], 0.5 mM K_3_[Fe (CN)_6_], 0.5 mM K_4_[Fe (CN)_6_], 10 mM EDTA, and 1% Triton X-100). The suspension was incubated in the dark at room temperature for 1 h. Following incubation, the mycelia were washed three times with 10mM PBS buffer (pH 7.2) to remove residual staining solution, and the mycelia were observed under a under an inverted microscope at 400× magnification in bright-field.

### 5.10. Statistical Analysis

Data are presented as the mean ± standard error of measurement (SEM). In the line graphs, SEM values are indicated by error bars. Statistical analysis was carried out using Student’s *t*-tests when applicable. The statistical results were derived from an independent and randomized experiment with three replicates, and a *p*-value < 0.05 was considered significant.

## Data Availability

Data will be made available upon request.

## Supplementary Materials

The following supporting information can be downloaded at: https://www.mdpi.com/article/10.3390/jof11090674/s1. Figure S1: The coding region sequence of the glycerol-3-phosphate dehydrogenase gene (*SrGPD*) from *S. rugosoannulata* HC7. Figure S2: Multiple sequence alignment of GPD protein sequences. Figure S3: Sequence alignment of *SrGPD* with the PLN02272 superfamily. Figure S4: Phylogenetic tree of GPD protein sequences from *S. rugosoannulata* and other selected fungi. Figure S5: The putative promoter sequence of the *GPD* in *S. rugosoannulata* HC7. Figure S6: Prediction of the *GPD* promoter elements in *S. rugosoannulata*. Figure S7: The putative terminator sequence of the *GPD* in *S. rugosoannulata* HC7. Figure S8: The coding region sequence of the succinate dehydrogenase [ubiquinone] iron-sulfur subunit (*SrSDI*) from *S. rugosoannulata* HC7. Figure S9: Alignment of transcripts obtained via full-length transcriptome sequencing against the reference sequence of the *SrSDI*. Figure S10: Phylogenetic tree of SDI protein sequences from *S. rugosoannulata* and other selected fungi. Figure S11: Sequence alignment of *SrSDI* with the PLN00129 superfamily. Figure S12: The putative promoter sequence of the *SDI* in *S. rugosoannulata* HC7. Figure S13: The putative terminator sequence of the *SDI* in *S. rugosoannulata* HC7. Figure S14: Schematic diagrams of the recombinant plasmids used for protoplast transformation in *S. rugosoannulata* HC7. Figure S15: The re-screening plates for *S. rugosoannulata* transformants obtained through Agrobacterium-mediated transformation with plasmid pCM-GHT-GGT (a), pCM-mSDI-GMT (b), or pCM-mSDI-GGT (c). Figure S16: PCR-based identification of the insertion fragments in *S. rugosoannulata* transformants generated using plasmids pCM-GHT-GGT (a), pCM-mSDI-GMT (b), and pCM-mSDI-GGT (c). Figure S17: Verification of exogenous DNA contamination in *S. rugosoannulata* transformants generated using the recombinant vectors pCM-GHT-GMT. Figure S18: Regeneration of *S. rugosoannulata* protoplasts. Figure S19: Schematic diagrams of recombinant plasmids used for constructing binary vectors. Table S1: The sequences of primers used in plasmids construction. Table S2: The primer sequences for the identification of transformants. Table S3: The primer sequences for mhiTAIL-PCR. Table S4: The GPD sequence information from 82 species was used for constructing the evolutionary tree in *S. rugosoannulata*. Table S5: The SDI sequence information from 87 species was used for constructing the evolutionary tree in *S. rugosoannulata*.
