# Supplementary material for: The Transformation and Protein Expression of the Edible Mushroom Stropharia rugosoannulata Protoplasts by Agrobacterium-tumefaciens-Mediated Transformation
Source: J Fungi (Basel). 2025 Sep 12;11(9):674. doi: 10.3390/jof11090674 (PMC12471233; doi:10.3390/jof11090674)
Supplement: Supplementary file 1 [file jof-11-00674-s001.zip › jof-3834839-supplementary-S1.pdf]

Supplementary File S1:

ATGgtatgtttgttcatgctgtcttgctggtgtttacgctcactgaactatctcagGTCAAAGTCGGCAT
TAACGGgtaagttcttcgcagcttgaggaacgattttgactgaggggccccaaatttgactagTTTCGg
tacgtttctacgagtttcggtttatgacggtgctaacgcaaaacagGTCGTATTGGTCGTATCGTCTTCC
GCAATGCTCTCGAGGTCCAGGGCATCGACGTTGTCGCCATCAATGAgtacgtcaatgtctagtcacattt
aggcgtaatcttatcacatactccagTCCCTTCATTGACCTCGACTACATGGTCTACATGTTCAAGTATG
ACTCCGTCCACGGCCGCTTCAAGGGCACCGTTGAGGCCAAGGATGGCAAGCTCGTCATTGACGGCAAGGC
CGTTACCGTCTTCGCTGAGCGTGATCCTGCTGCGATTCCTTGGGAACCGCCGGCGCTGACTATGTCGTT
GAGGCTACTgtgagctatcttcatctcgtatttcgcccacgcaatctcatcgcttctgccgggaacagGG
TGTCTTCACCACCACCGACAAgtgagcattacgtcgtcattgctctatctcgagtatgttgacagtttt
cctagGGCTTCTGCCCACTTGAAGGGCGGTGCCAAGAAGGTCATTATCTCCGCGCCTTCTGCGGATGCCC
CCATGTTCGTCTGCGGTGTCAACTTGGACGCTTACGATCCCAAGTACCAAGTCgtaagttgctccgccat
cgcaatctatgcgagatctgatgtgtgaattgttacgtcatttatgcgattagATCTCCAACGCTTCGTG
CACCACCAACTGCCTTGCGCCCCTGGCCAAGATCATCCACGACAAGTTCGGCATTGTTGAGGGCTTGATG
ACCACCGTCCACGCCACCACCGCCACCCAAAAGACTGTCGATGGACCCTCCCACAAGGACTGGCGTGGAG
GACGCTCCGTCAACAACAACATCATTCCCTCTTCCACTGGTGCCGCCAAGGCCGTCGGAAAGGTCATTCC
TTCGCTGAACGGCAAGCTCACgtacgtgcttttcaagccatactatctggagaataggctaacgtatatc
tatacagCGGTCTTGCGTTCCGTGTCCCCACTCTTGATGTCTCCGTTGTCGATCTCGTTGTCCGCTTGGA
GAAGGGAACTTCCTACGACGATATCAAGGCTGCCGTCAAGGCCGCCTCTGAGGGTGACTACAAGGGCATT
GTCGGCTACACCGAGGACCAGGTCGTCTCCACCGACTTCATCGGCAACCACAACTCGTCAATCTTCGACG
CCAACGCCGGAATCCAGCTGAGCCCCAACTTCGTCAAGCTGATCTCCTGGTACGACAACGAGTGGGGTTA
CTCCCGTCGTGTCTGCGACCTCATCAACTACGTCGCTGCTAAGGATGCCGCCGCTGGCATTTAA

Fig. S1 The coding region sequence of the glycerol-3-phosphate dehydrogenase gene (*SrGPD*) from *S. rugosoannulata* HC7. Uppercase letters indicate exons, while lowercase letters indicate introns.

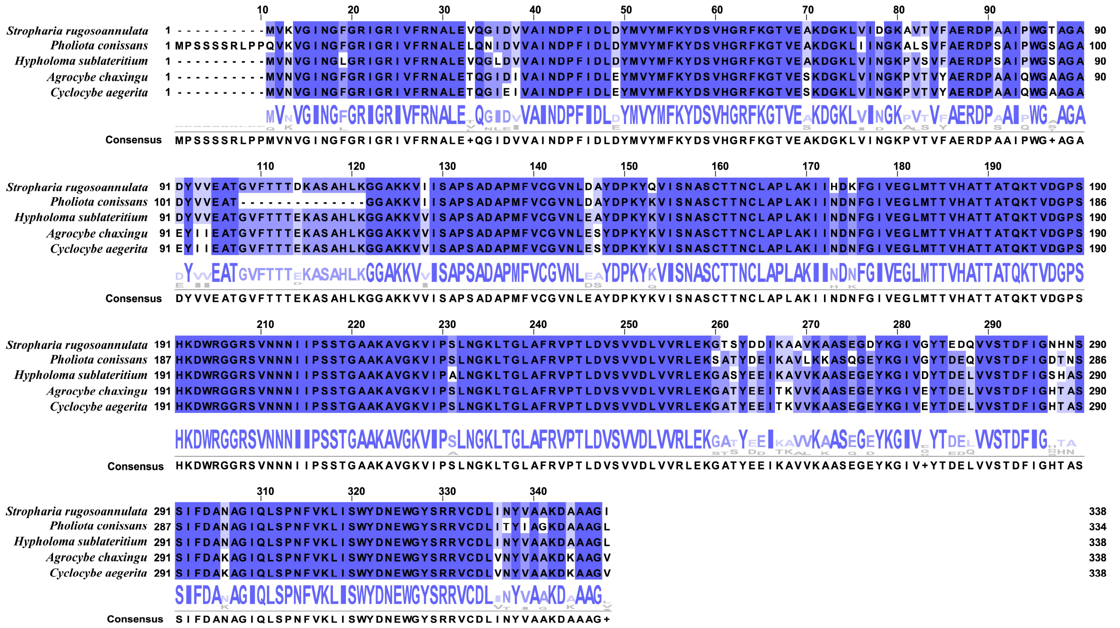

Fig. S2 Multiple sequence alignment of GPD protein sequences. The multiple sequence alignment results comparing the SrGPD protein sequence with GPD protein sequences from other species are presented.

```
                   *        20         *        40         *        60         *        80
PLN02272 : MAFSSLLRSAATAPAAAARGSDFSSSSSDPSKVSSVGFSSSLSFSGSSSGASSSLQSCSARSVQPIKATATEAPPAVLKS :  80
SrGPD    : -------------------------------------------------------------------------------- :   -

                   *       100         *       120         *       140         *       160
PLN02272 : SSSGKTKIGINGFGRIGRLVLRIATSRDDIEVVAVNDPFIDAKYMAYMFKYDSTHGNFKGTINVVDDSTLEINGKQIKVT : 160
SrGPD    : ----MVKVGINGFGRIGRIVFRNALEVQGIDVVAINDPFILLDYMVYMFKYDSVHGRFKGTVEAKDG-KLVIDGKAVTVF :  75
               K GINGFGRIGR V R A     I VVA NDPFID  YM YMFKYDS HG FKGT     D  L I GK   V

                   *       180         *       200         *       220         *       240
PLN02272 : SKRDPAEIPWGDFGAEYVVESSGVFTTVEKASAHLKGGAKKVVISAPSADAPMFVVGVNEKTYKENMNIVSNASCTTNCL : 240
SrGPD    : AERDPAAIPWGTAGADYVVEATGVFTTTDKASAHLKGGAKKVIISAPSADAPMFVCGVNLDAYDPKYQVISNASCTTNCL : 155
             RDPA IPWG  GA YVVE  GVFTT  KASAHLKGGAKKV ISAPSADAPMFV GVN    Y P     SNASCTTNCL

                   *       260         *       280         *       300         *       320
PLN02272 : APLAKVVHEEFGILEGLMTTVHATTATQKTVDGPSMKDWRGGRGASQNIIPSSTGAAKAVGKVLPELNGKLTGMAFRVPT : 320
SrGPD    : APLAKIIHDKFGIVEGLMTTVHATTATQKTVDGPSHKDWRGGRSVNNNIIPSSTGAAKAVGKVIPSLNGKLTGIAFRVPT : 235
           APLAK  H   FGI EGLMTTVHATTATQKTVDGPS KDWRGGR    NIIPSSTGAAKAVGKV P LNGKLTG AFRVPT

                   *       340         *       360         *       380         *       400
PLN02272 : PNVSVVDLTCRLEKSASYEDVKAAIKYASEGPLKGILGYTDEDVVSNDFVGDSRSSIFDAKAGIGLSASFMKLVSWYDNE : 400
SrGPD    : LDVSVVDLVVRLEKGTSYDDIKAAVKAASEGDYKGIVGYTEDQVVSTDFIGNHNSSIFDANAGIQLSPNFVKLISWYDNE : 315
             VSVVDL  RLEK   SY D  KAA K ASEG   KGI GYT   VVS DF G    SSIFDA AGI LS   F KL SWYDNE

                   *       420
PLN02272 : WGYSNRVLDLIEHMALVAASH-- : 421
SrGPD    : WGYSRRVCDLINYVAAKDAAAGI : 338
           WGYS RV DLI   A   A
```

Fig. S3 Sequence alignment of *SrGPD* with the PLN02272 superfamily. Amino acids that are identical in the alignment results are highlighted with a black background.

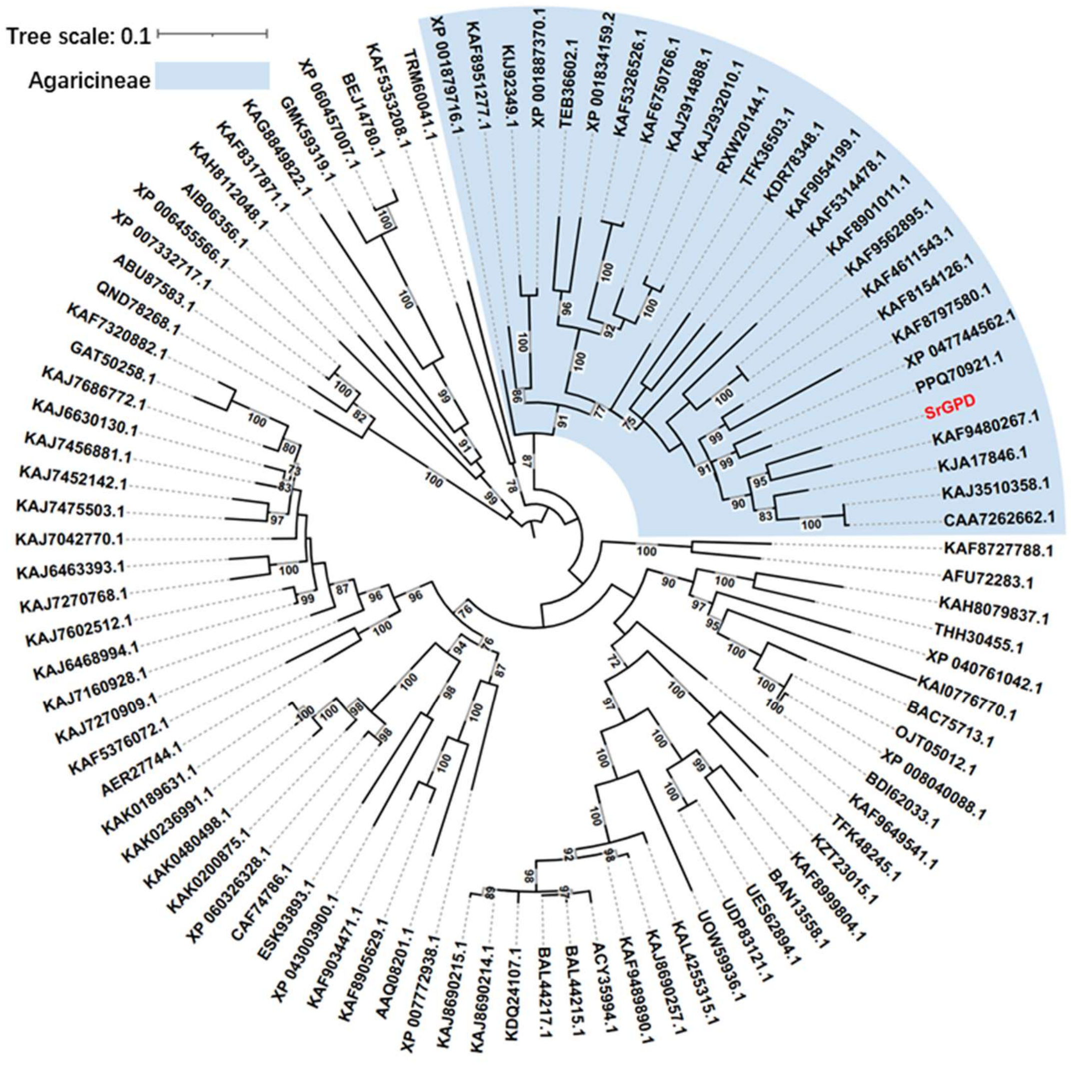

Fig. S4 Phylogenetic tree of GPD protein sequences from *S. rugosoannulata* and other selected fungi. The sequences used for constructing the phylogenetic tree included the SrGPD protein sequence and 94 GPD protein sequences derived from 82 fungal species (The correspondence between the sequence accession numbers and their respective species is detailed in Table S4). The tree is midpoint rooted and is drawn to scale with branch lengths computed using the Maximum Likelihood method with 10,000 ultrafast bootstrap replicates and measured in the number of expected substitutions per site. the ultrafast Bootstrap values which more than 70% are shown above or below the branches. The species corresponding to accession number which covered by the blue background all belong to Agaricineae.

```
accgtccattccacgacggcgacacagaaaaccgtggacggcccatctcacaaggactggcgtggggtc
gcggagtcggtaccaacattattccttcatcaacgggagctgcgaaggccgtaggaaaagtcataccgtc
ccttaatggaaagttaacgtaagtagaatgttttaattggtaacgaagaaaataaacttaacagcattta
cttagcggtcttgcgttccgcgttccgacaatcgatgtctccgttgtggatctcgtcgttcgccttgaga
aaggtgccaagtatgacgatatcaaagcgacggtgaaggcagcgtcccagagtaaagaatacgccgggat
tataggttataccgatgagcaagttgtatcaaccgatttcattggaagcgactattcttccatcttcgat
gcgctcgccggaatacagctcaacgataacttcgtcaagttgatcgcctggtacgataacgaatggggat
actcccaccgtgtctgcgatttggtggcttttgtcgctgctgccgacaagaaataggcgacttgacgagt
aagtagatgacctactcacattctagatcctgcaaaaacgcccgttttggtcttttcattgtccagattt
ctatggtagattcgaaccacactgtatactatgcatcacacaacagcaatgatattcaataatttcgagt
tagtgtcccacccggcctctaaaaacgtcataagtaatgtccgttgtccgttgaggttaattaggaaggc
cttcggatctggcccctttccttaatgagttatttttaggcatctctctgaacaggagtctagactgtcg
cgaggcgcctatgcacaggcagacatggacagactccgcctgtgcacaggtcgcggttgggtcgcggata
atcgtcgtcgctaatacgcatgtgcggtctaggtgcggtcattgtttctagtgcgtacgagtatgtgtta
atttcatttcaatagaaacgttcatcggttttgctttgtagtcctgctttatcggaaatgtacgagagac
tcctgaatcggcttggtgtggcacagcaaggcgcggatgtcggtgtgcctcagcaacatttgtgaatatt
cgaacagctcgacttgttctgggattgataatggattactcattgctgacggtccagctcttacttttgg
cggttgtttatagtgcagtactggaatagcaatggaatgcaacgagatggagcgcgcctcggcctagagc
tataggccgaacgaaggctttccttttgcagccaatggggaccaaaccagttcagggcggccgatattct
ggttgctgacccaactcggcgccgcgcaatgtgattggacgtcatatactctcatcgatagataatcttt
ctggcgggactatataagttgggccgccgtcggactttcgctcctcgcgcattttgcattcaacctcta
cccaccacatccaccaccatcttaatcacc
```

Fig. S5 The putative promoter sequence of the *GPD* in *S. rugosoannulata* HC7.

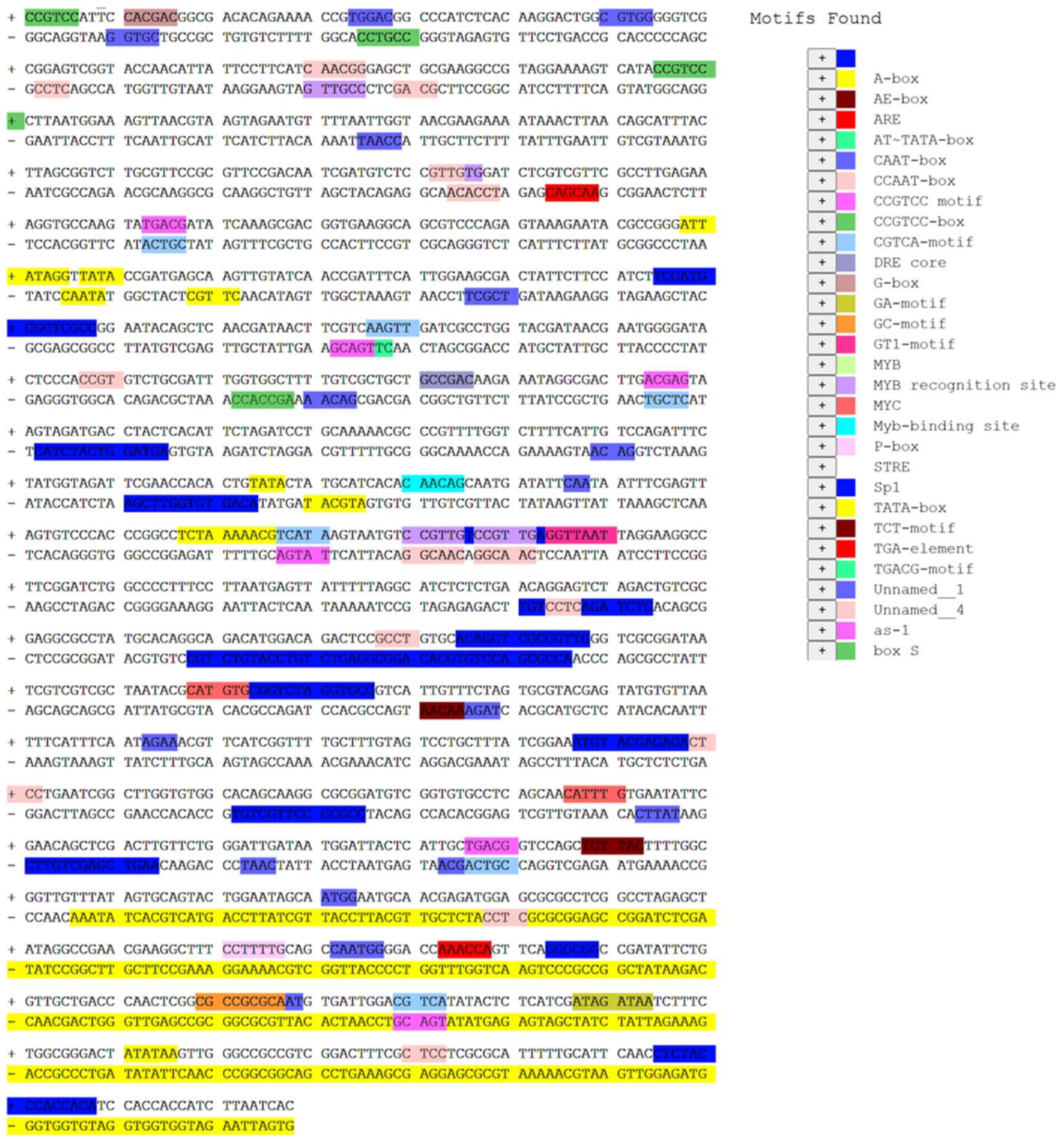

Fig. S6 Prediction of the *GPD* promoter elements in *S. rugosoannulata*. The PlantCARE online tool was employed to identify motifs within the putative *GPD* promoter region of *S. rugosoannulata*. Different base background colors indicate the distinct motifs that have been predicted.

gtgttctctgagtttcaggggtaccgagtcaaaattaatgaaagatatagcagaatttatgtgtgtaatt
ctatagagagtatcttgtacaatatacatacatcttggactgtatgcgccagcaaccatcttgcgtactt
tggagtatttttcaaccatccaagatcataaatttaaaatacgtttgaagtcgaacaagaattcgttatc
atgaaagacactttggcagacgcaccgagtagatagactacacagagcgcatgggagaagaagggtggaa
gaatggtgaaaaaaaacgaaatagagaaggtggtactcgggatgcttgccgagacgttgacgtttctccg
ccgcaaccaccgcacgtctacggcaagtctacggctgtttcaacagcgtacgagagtttgctgtgttggt
tcacccgcagcttcgcgcggctcgataccactcgatagcaatatccaatttaccgtcaccgataccgagc
acgttcgttatccagtcgataaggatcccgtctccgatgactcaggttcgacggtggtaacggtttgcta
ccgtatgctcaagtgaaaatttatttcaacgcatgcgcagtctcgtctgaccaatggactactgcctta
ttcaccaatgtggacatcgagtgtggcatttagtgcgaccttgtcggtgtacaggaagggacagtccgac
atgtcacggcaattaaatgcacattcgatagagaagcaaatatgatacaagcacgaatgtaaatgctgcc
tgcggtctcatccctgcctactttctttatttaggggaaagggtatgtggttagactatagggatgcc
atgggtatattgttcagagtgcgcgctactgggaatggcgagcgtgtcgtgcgcgtcagcgtgcgctctg
agctttgatgcatttgtggcacggatcgcattgactataatttacattttcgtgggtttacactaataa
ttcatattaatcacatactt

Fig. S7 The putative terminator sequence of the *GPD* in *S. rugosoannulata* HC7.

ATGCAGTCAGTTCTTGCGCAGGCTCGCCTCAACATCGGCAGGCGCGTTTTCTACCTCCGTCGCGAGAT
GGCAGGCAGTGCCCCAGCAGAAACCAGTCTTGATGAAGGAGTTTAAGATCTACCGCTGGgtacgctcacg
cacattcagttcagggttctcctgggagtctaaccccaagttaacagAATCCTGATGAGCCTGAGAAGAA
GCCTGAGCTGCAGTCGTATAAGATCGATCTGAACCAGACGGGACCCATGgttagacttctcgacttctgt
ttctagcatcgttttcaacagtcatttgggcgtttttcagGTACTCGACGCCCTTATCAAAATCAAGAAT
GAGGTCGACCCTACGCTCACCTTCCGTCGTTCTTGCCGTGAAGGCATTTGCGGTTCATGCGCTATGAACA
TCAACGGACAGAACACTCTTGCTTGCTTGTGCAGGATTGACCGGGACGGCAGCTCGGACGCCAAAATCTA
CCCTCTTCCACATAgtgagtatctttattcgattcaaacaagcaggggctgactgtctcactttagTGTA
TGTTGTTAAGGACCTCGTCCCGGACATGACTCTCTTCTATAAGCAATACAAGTCCATCCAGCCTTGGCTG
CAAAACGAGAACCCCCCAGCACAGGgtatggattgccatgttcagattcaaatattttcttattatccaa
ctctatagGCGAGTTCCTGCAATCCCCAGAAGACCGTCGCAAGCTTGACGGCATGTACGAATGCATTCTT
TGCGCTTGCTGCTCTACTTCCTGCCCATCGTACTGGTGGAATCAGGATGGGTACCTTGGTCCTGCGACAT
TAATGCAGGCGTACCGATGGATCGCTGACTCTCGGgtatgtactttcagcttgacggggataaagcggaa
cttaacatatttcaacgaacatacagGACAGCCACGGTGCGGAGCGAATGGAGAAGCTGCAGAACGAAAT
GAGCTTGTACCGTTGCCATACCATCTTTAACTgtctgtatttccgctgctactgctgctttgattgtgtg
tttgctcactggctcatcttgcagGTTCGCGCACCTGCCCCAAGGGTCTTAACCCTGCCGCAGCCATCGC
TAAGATCAAACTAGAAATGGCCATGGATTAA

Fig. S8 The coding region sequence of the succinate dehydrogenase [ubiquinone] iron-sulfur subunit (*SrSDI*) from *S. rugosoannulata* HC7. Uppercase letters indicate exons, while lowercase letters indicate introns.

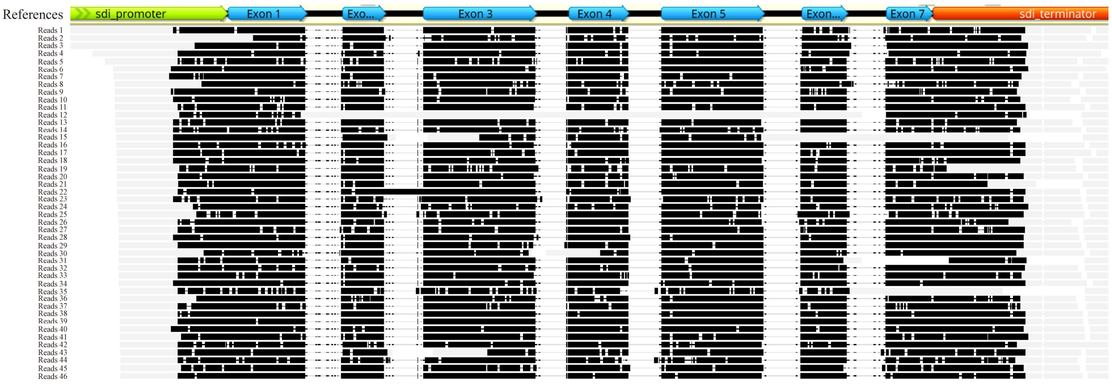

Fig. S9 Alignment of transcripts obtained via full-length transcriptome sequencing against the reference sequence of the *SrSDI*. The transcript sequence (indicated by black boxes) is largely consistent with the exon regions (marked in blue) of the reference

sequence.

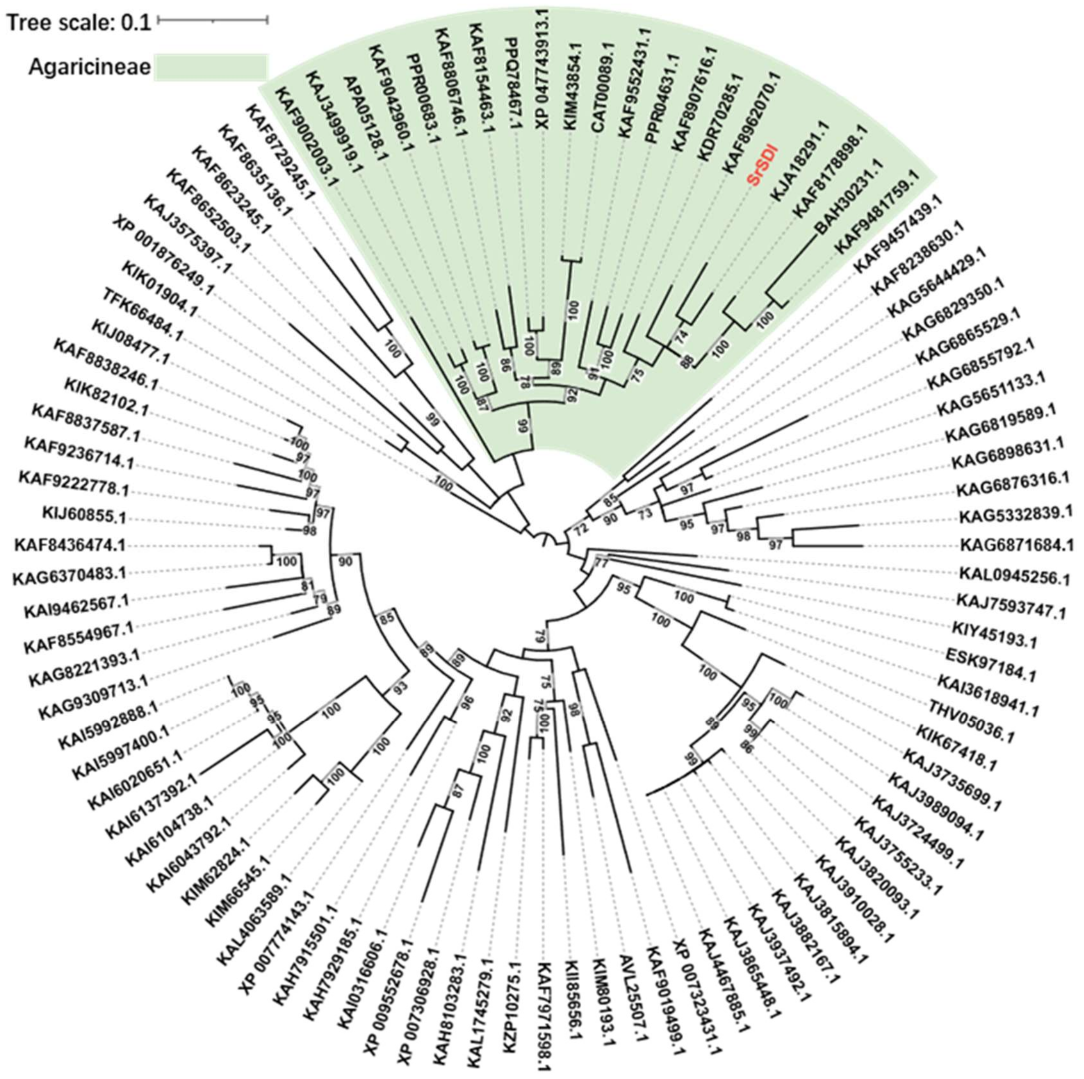

Fig. S10 Phylogenetic tree of SDI protein sequences from *S. rugosoannulata* and other selected fungi. The sequences used for constructing the phylogenetic tree included the SrSDI protein sequence and 95 SDI protein sequences derived from 87 fungal species (The correspondence between the sequence accession numbers and their respective species is detailed in Table S5). The tree is midpoint rooted and is drawn to scale with branch lengths computed using the Maximum Likelihood method with 10,000 ultrafast bootstrap replicates and measured in the number of expected substitutions per site. the ultrafast Bootstrap values which more than 70% are shown above or below the branches. The species corresponding to accession number which covered by the blue background all belong to Agaricineae.

```
                     *        20         *        40         *        60         *
PLN00129 : MAAGLLRRLAGAKAGLLAPAAAASAAASAETKASSKGSKPSNLKEFQIYRWNPDNPGK-PHLQSYKVDLND :  70
SrSDI    : ----MQSVLAQARLNIGRRAFSTSVARWQAVPQQ----KPVLMKEFKIYRWNPDEPEKKPELQSYKIDLNQ :  63
                   LA A       A   S A           KP   KEF IYRWNPD P K P LQSYK DLN

                 80         *       100         *       120         *       140
PLN00129 : CGPMVLDVLIKIKNEQDPSLTFRRSCREGICGSCAMNIDGKNTLACLTKIDRDESGPTTITPLPHMFVIKD : 141
SrSDI    : TGPMVLDALIKIKNEVDPTLTFRRSCREGICGSCAMNINGQNTLACLCRIDRDGSSDAKIYPLPHMYVVKD : 134
            GPMVLD LIKIKNE DP LTFRRSCREGICGSCAMNI G NTLACL  IDRD S    I PLPHM V KD

                 *       160         *       180         *       200         *
PLN00129 : LVVDMTNFYQQYKSIEPWLKTKKPPEDGQKEHLQSKEDRAKLDGMYECILCACCSTSCPSYWWNPEKFLGP : 212
SrSDI    : LVPDMTLFYKQYKSIQPWLQNENPPAQG--EFLQSPEDRRKLDGMYECILCACCSTSCPSYWWNQDGYLGP : 203
           LV DMT FY QYKSI PWL    PP  G  E LQS EDR KLDGMYECILCACCSTSCPSYWWN    LGP

               220         *       240         *       260         *
PLN00129 : AALLHAYRWISDSRDEYTKERLEALDDEFKLYRCHTIRNCSNACPKGLNPAKAIAKIKQLLGLG : 276
SrSDI    : ATLMQAYRWIADSRDSHGAERMEKLQNEMSLYRCHTIFNCSRTCPKGLNPAAAIAKIKLEMAMD : 267
           A L  AYRWI DSRD    ER E L  E  LYRCHTI NCS  CPKGLNPA AIAKIK
```

Fig. S11 Sequence alignment of *SrSDI* with the PLN00129 superfamily. Amino acids that are identical in the alignment results are highlighted with a black background.

```
gtaacaagaatgcaggttatggcaatgctccagtattaattagaggatttgctgcatgttggaacttgga
agtgtagtttggtgagttattagcgtgtcggtgcaaatgtatgcaacttgtaacttgggtccgacagaga
aatcgcggttcgtgaacctaacaaatgtaaagggatcagttatatgacgcagacataacacctggtccta
taagtcatttgtaggtcgtataaaaaacaggttgtagacatggtcataaactgttagttgattcaaggcg
aatgctgtccttggcgttactcagaaaggtatctaaaacattggatgaacgtagatggaggaaacaagtc
agaaatgtcgtccaagaagtgaagcacgccaagtagatttacaggaaatgggttatcatattgtcgcgag
cagggatcgagaatgtcaagatgcatctcgcgcactagataattccagtaggctctgatattggcaccat
cgcaaatgttcacgtccagtcgcttggtccaccactcgaggtaggcacggagaaatcgtaccaggagagg
acgattgatgggcaccttcaccatctaagataatatgtgccctcgtccaaggatgacgtactcgtacaga
atcggcactgttagggagtaccaggtctagttcactctaatgaatttgcttcttgtacctgaacgctggg
cagtactatgcagtcagatttgttgctaactcttaagtcttctttgaggacctcaccactactgttcgct
gctgtcgaagacagggcttttccaatgaggtcccagggccaaagtacagtgatatgatatcgagctcaag
ccctcgaggaggcaaggaatagcgtttctagagccattcaattggcccgcaaagtttctgtggaaaagaa
agtccttgaaaatcgtcagcttggcctgcggaaaaggcgtgtttgtcgctacaaaatcgattattggagc
cggccatgtggtatatgcgtgatcaagactttgggcagaaccaggcaaacacagagtgtggattgcgttg
tgtttccttaggtcgaggtatgcttcaggttactcgagatatatcatttaacgtcctggatttgtgtttg
atccgtagatgacattcaaaaggtattgaggaatcgtcgcagtccttctgcagatacatggggtaaccct
ttgtcacagtcatttgaagtgtgtagttggttcaacgaggtttaagtttgatcctgtagaacaagtatta
ctgtaatcgtatgcttgatttggatttttgtggatatatgcgcatattctgtgaataaatacgcttattat
gagaatatatgcgcctattagttcgttgaataggcagacatgtcggactggtagacgttgggtagaggat
gactttcaggtggactgcgattgggtggagagtcggctatttgtggcaaacatccggtatcacgcgacaa
acgggattcggtccccacctttcctgccaacacccaagtccatttccgtatttctccaccgctgccatc
ctcagtgtgtttcccctata
```

Fig. S12 The putative promoter sequence of the *SDI* in S. rugosoannulata HC7.

aaggtgtagctgcaaggaaggcgcattggctgtaattaagcaaaggcgatacggcaagttagacaatgca
cataactattccttcaaatagcagaggatactatggcatttgattgtataaaagtaatttttttaaaact
ttttatttttctggagcgtgtacttctaagttgcactttcgatcgcctccgcagtcttcgcaacggttct
tgaatgaacgcgaccacagtcctttccaggcggcgacgtagtcgactcgcggacatcatggatcacaccg
gataattatccgtgccgatccgggtgttaaaaacacaatgtgttctctgcttgttctatgccaattcgct
tcttgactcgcaaccaccatgactacctttgccaaatcgacgtttaatgcttctgtttattctgcttcac
gtccgacatactcttctcaactcttcgactatatctttgacttccatcgcaggggaggtacaggcagcgc
cacctggcaccgtgccgtagatgtaggatgcggaacaggtacttccacagaagagctcactacgtctgct
aaacaaagcgtcaggtcaagctactgcgcatctacgaggtttccgtgaagttattgccgtggatccatct
gcagttatgctagacaaggcaaaggtgtacttagaaaacaacgattccagtactcttggtcccaaattta
ccttctcgcaaggttcagcagaagatctgagacaggcagtgccggaagatgagagcgtggatttacttgt
agctggtactttgcttcaacctcctcgactgctgagtactcattcgcgaaatataatttattagctcaag
ctgcgcactggtttgattggagcaaggtctggccggagacctctcgtgtcctcaagaaaaacggcactgt
cgctttctgggtttgtgataggcactctttctgaccgcaacgatttttatgacacgcatatcttatcacct
atgaataggtctacgcggagtttcgacttaccaggtacccctccctc

Fig. S13 The putative terminator sequence of the *SDI* in *S. rugosoannulata* HC7.

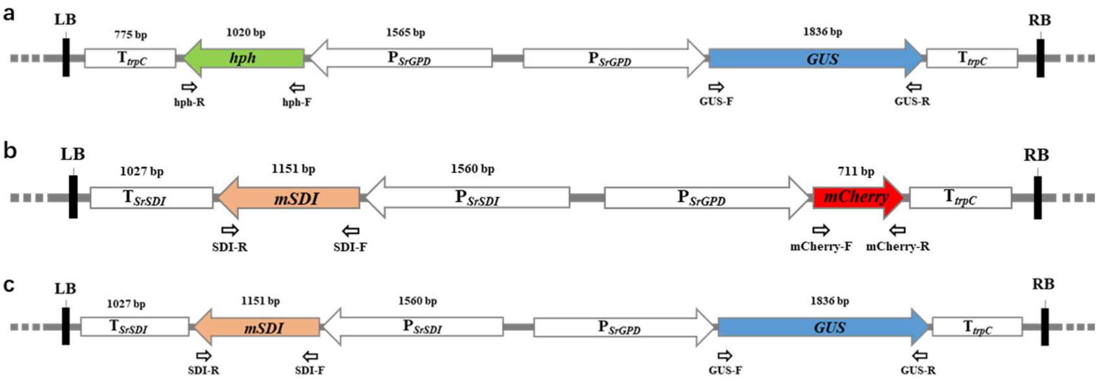

Fig. S14 Schematic diagrams of the recombinant plasmids used for protoplast transformation in *S. rugosoannulata* HC7. a The recombinant plasmid pCM-GHT-GGT. **b** The recombinant plasmid pCM-mSDI-GMT. **c** The recombinant plasmid pCM-mSDI-GGT.

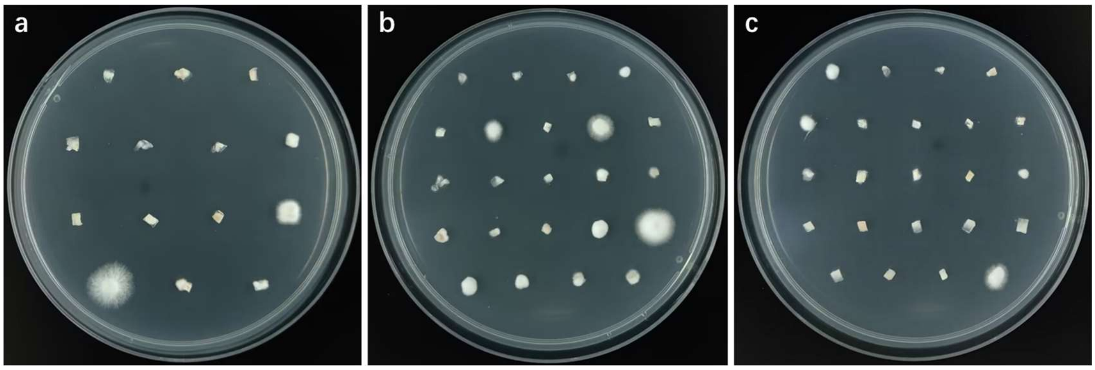

Fig. S15 The re-screening plates for S. rugosoannulata transformants obtained through Agrobacterium-mediated transformation with plasmid pCM-GHT-GGT (a), pCM-mSDI-GMT (b), or pCM-mSDI-GGT (c).

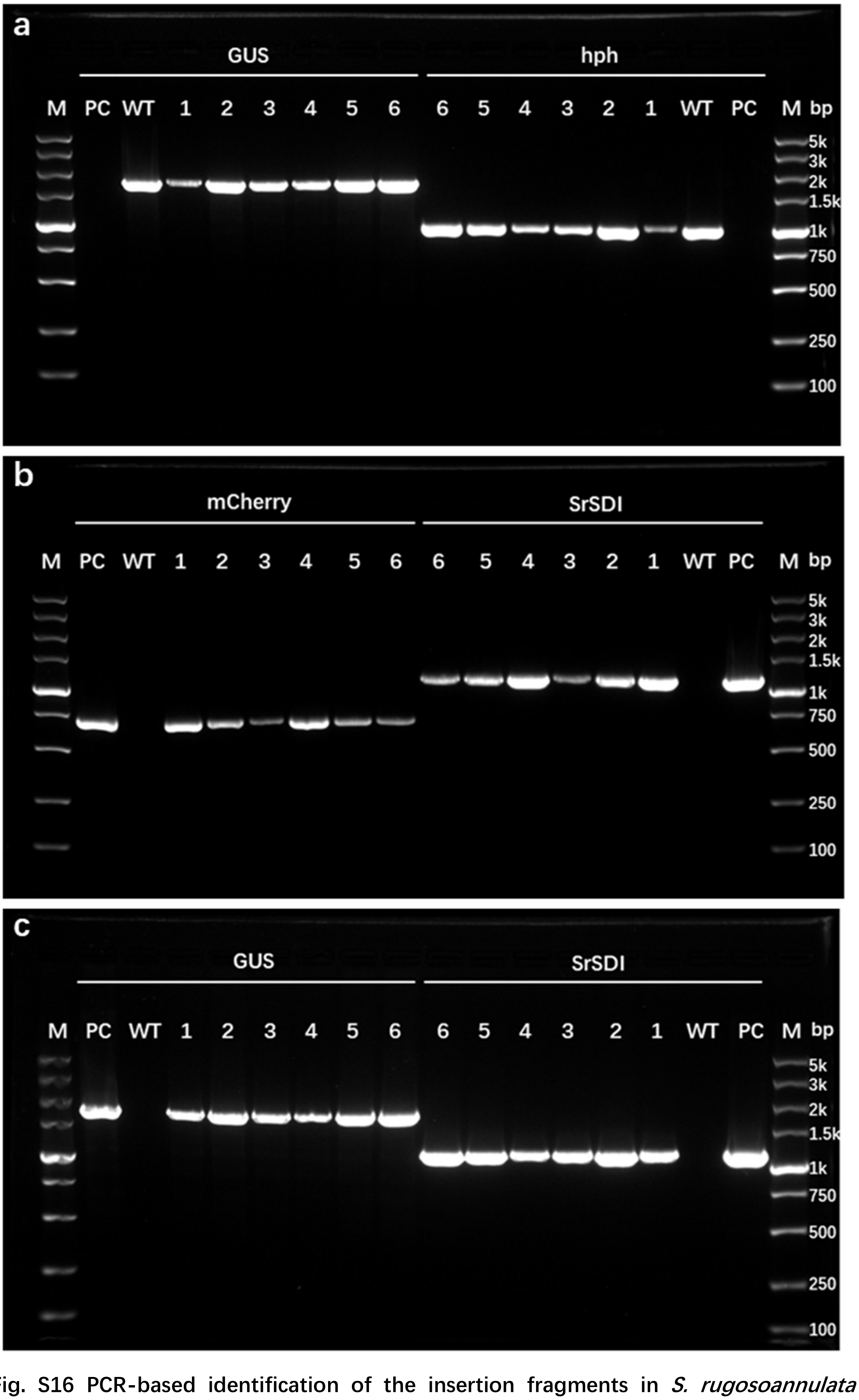

Fig. S16 PCR-based identification of the insertion fragments in *S. rugosoannulata*

transformants generated using plasmids pCM-GHT-GGT (a), pCM-mSDI-GMT (b), and pCM-mSDI-GGT (c). The primers employed are those depicted in Fig. S14. PC, plasmid positive control. WT, wild type. 1-6, transformants after re-screening. M, DNA molecular marker.

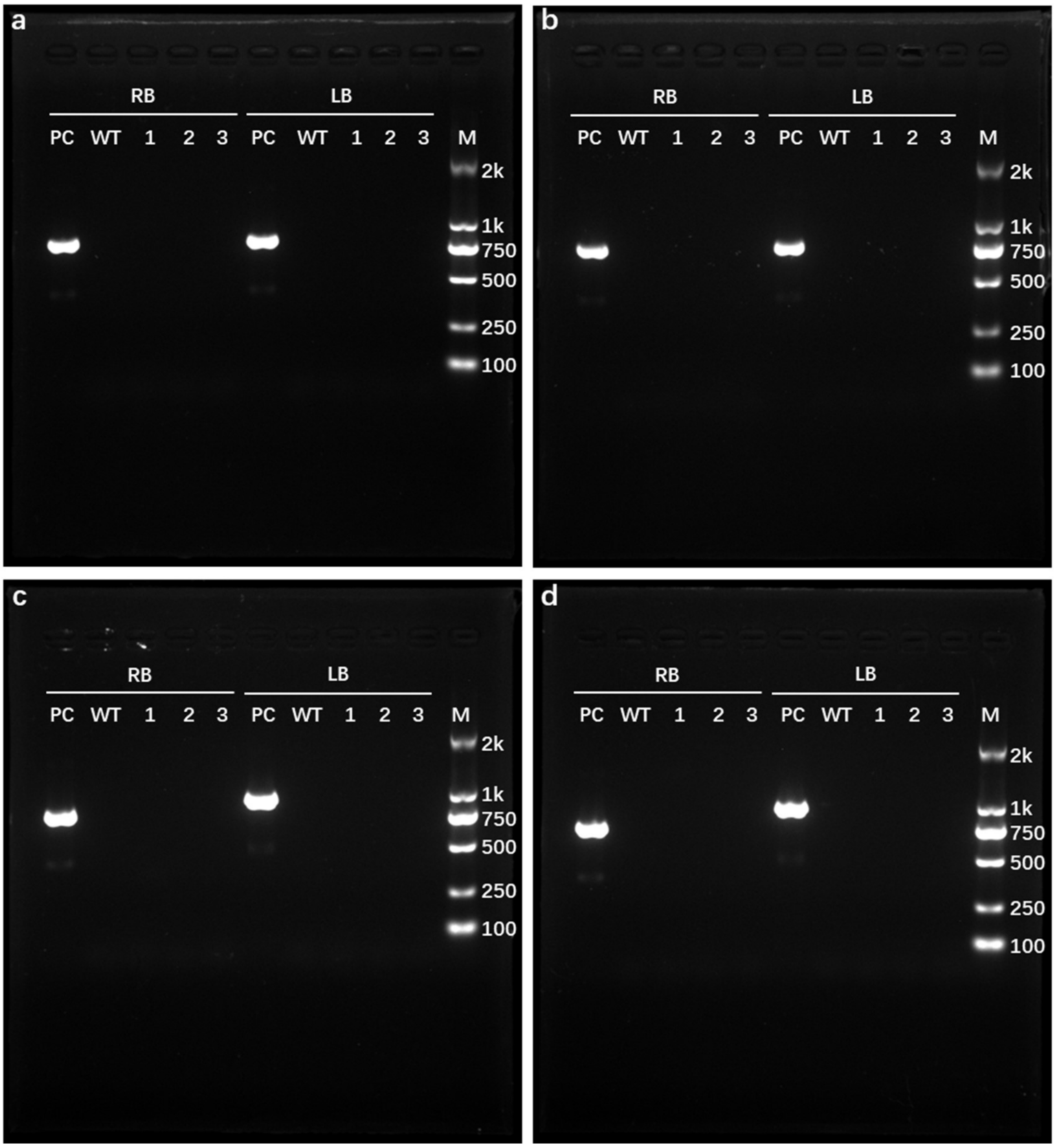

Fig. S17 Verification of exogenous DNA contamination in *S. rugosoannulata* transformants generated using the recombinant vectors pCM-GHT-GMT (a), pCM-GHT-GGT (b), pCM-mSDI-GMT (c), and pCM-mSDI-GGT (d). The right border sequence (RB) of the plasmid insertion fragment was validated using the primer pair LPU-R/RPU-R. The left border sequence (LB) was validated using the primer pairs LPU-F/LPU-R (for plasmids pCM-GHT-GMT and pCM-GHT-GGT) or LPU-F/LPUS-R (for plasmids pCM-mSDI-GMT and pCM-mSDI-GGT). PC, plasmid positive control. WT, wild type, used as a negative control to exclude non-specific binding of genomic DNA to the primers. 1-3: re-screened *S. rugosoannulata* transformants. M, DNA molecular marker.

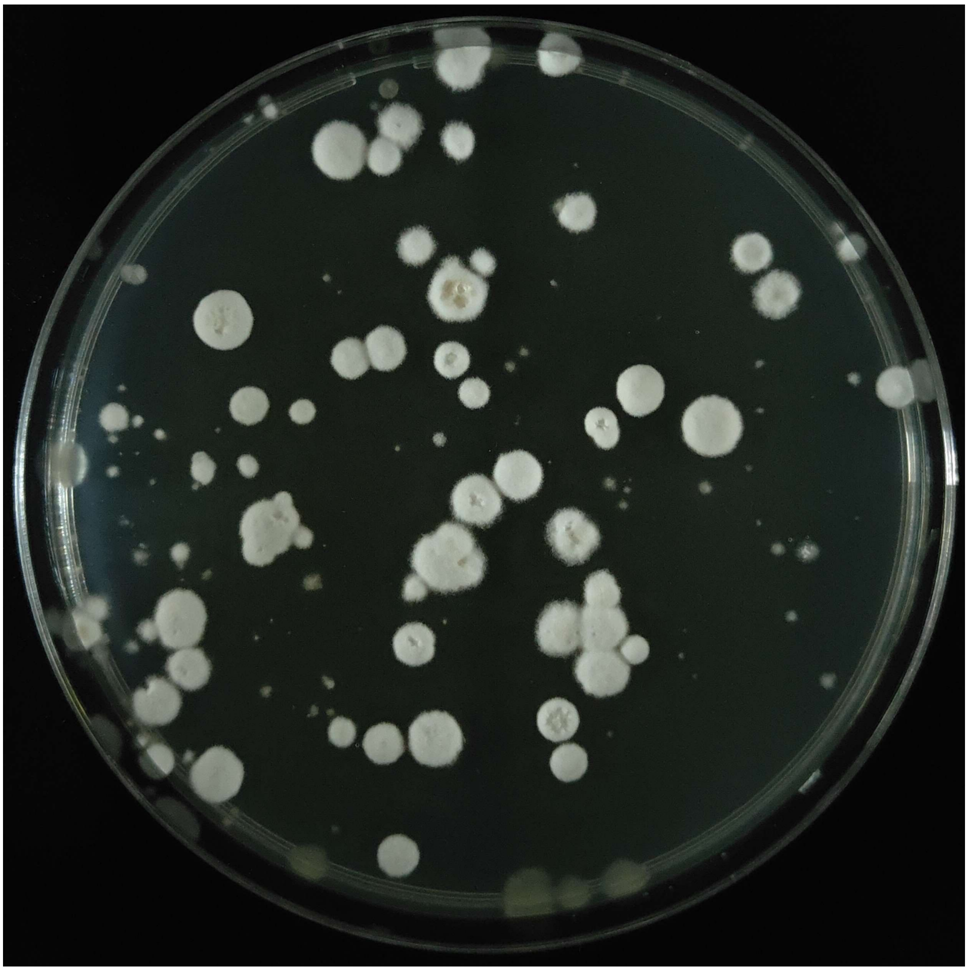

Fig. S18 Regeneration of *S. rugosoannulata* protoplasts. The figure shows the regeneration status of 10^4 protoplasts cultured on the regeneration medium for 10 days at 25°C.

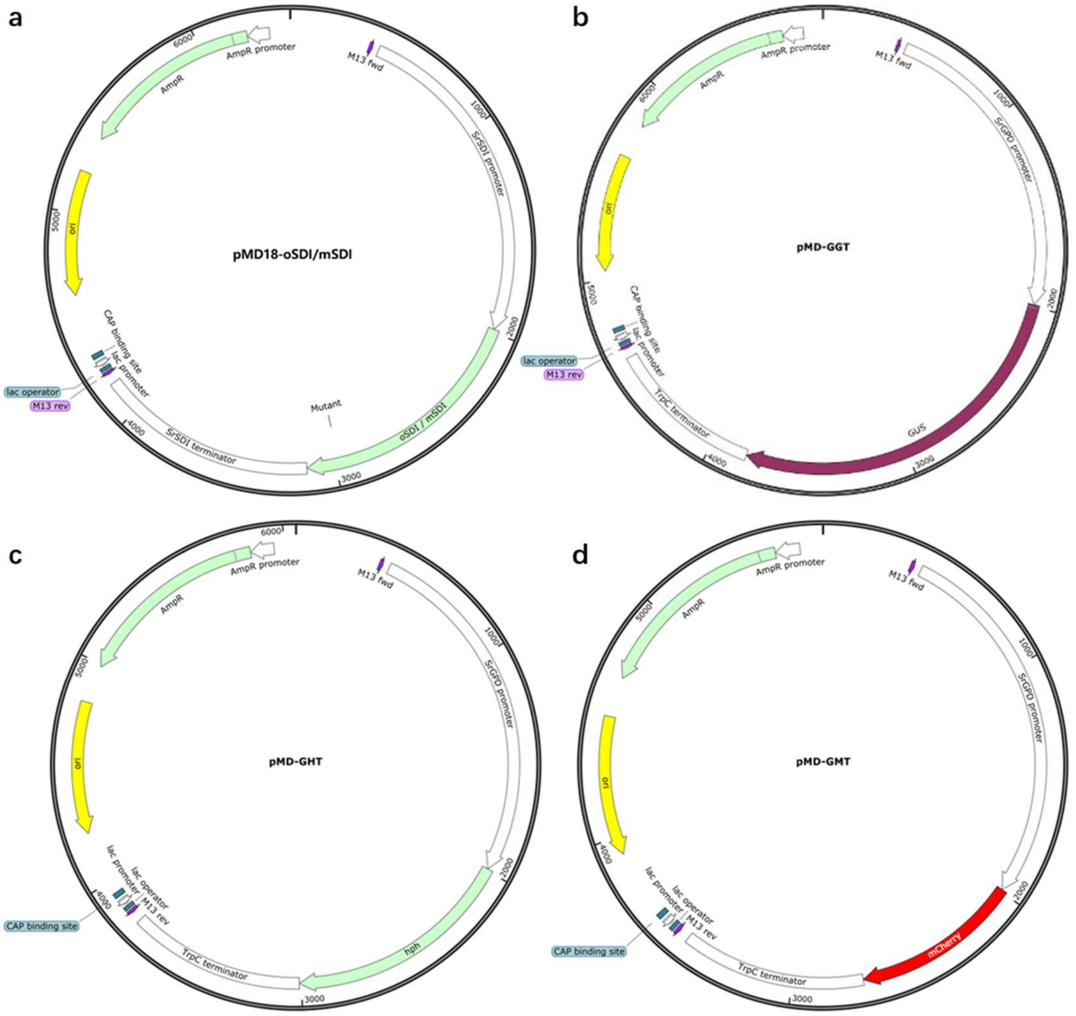

Fig. S19 Schematic diagrams of recombinant plasmids used for constructing binary vectors. a Plasmid maps of pMD-oSDI or pMD-mSDI. **b** Plasmid map of pMD-GGT. **c** Plasmid map of pMD-GHT. **d** Plasmid map of pMD-GMT.
