# Supplementary material for: The Transformation and Protein Expression of the Edible Mushroom Stropharia rugosoannulata Protoplasts by Agrobacterium-tumefaciens-Mediated Transformation
Source: J Fungi (Basel). 2025 Sep 12;11(9):674. doi: 10.3390/jof11090674 (PMC12471233; doi:10.3390/jof11090674)
Supplement: Supplementary file 1 [file jof-11-00674-s001.zip › jof-3834839-supplementary-S2.pdf]

Table S1 The sequences of primers used in plasmids construction.

Number	Primer name	Primer sequence (5′ to 3′)
1	hph-F	ATGCCTGAACTCACCGCGAC
2	hph-R	CTATTCCTTTGCCCTCGGACG
3	mCherry-F	ATGGTGAGCAAGGGCGAG
4	mCherry-R	CTACTTGTACAGCTCGTCCATGC
5	GUS-F	ATGGTAGATCTGAGGGTAAATTTCTAG
6	GUS-R	TTGTTTGCCTCCCTGCTG
7	TtrpC-F	GATCCACTTAACGTTACTGAAATCATC
8	TtrpC-R	TCGAGTGGAGATGTGGAGTG
9	GPDi-F	ACCGTCCATTCCACGACGG
10	GPDi-R	GACTTTGACCTGAGATAGTTCAGTGAGC
11	SDI-F	GTAACAAGAATGCAGGTTATGGCAATG
12	SDI-R	GAGGGAGGGGTACCTGGTAAGTCG
13	mut-SDI-F	GCTTGTACCGTTGCCTTACCATCTTTAACTG
14	mut-SDI-R	CAGTTAAAGATGGTAAGGCAACGGTACAAGC
15	pCM-F	AAGCTTGGCACTGGCCG
16	pCM-R	CCCCGAATTAATTCGGCGTTAATTC
17	SP-F	ATAGCCCTTTGGTCTTCTGAG
18	SP-R	CTGCAGGTCGACTCTAGAGG
19	homo-GH	GAACTATCTCAGGTCAAAGTCATGCCTGAACTCACCGCGAC
20	homo-HT	TCAGTAACGTTAAGTGGATCCTATTCCTTTGCCCTCGGACG
21	homo-GM	GAACTATCTCAGGTCAAAGTCATGGTGAGCAAGGGCGAG
22	homo-MT	TCAGTAACGTTAAGTGGATCCTACTTGTACAGCTCGTCCATGC
23	homo-GG	GAACTATCTCAGGTCAAAGTCATGGTAGATCTGAGGGTAAATTTC
24	homo-GT	TCAGTAACGTTAAGTGGATCTTGTTTGCCTCCCTGCTG
25	homo-GTS	CTCAGAAGACCAAAGGGCTATACCGTCCATTCCACGACGG
26	homo-BGT	TTAACGCCGAATTAATTCGGGGTCGAGTGGAGATGTGGAGTGG
27	homo-SGT	CCTCTAGAGTCGACCTGCAGACCGTCCATTCCACGACGG
28	homo-GTB	ACGACGGCCAGTGCCAAGCTTTCGAGTGGAGATGTGGAGTGG
29	homo-BMS	TAACGCCGAATTAATTCGGGGGAGGGAGGGGTACCTGGTAAG
30	homo-MSS	CTCAGAAGACCAAAGGGCTATGTAACAAGAATGCAGGTTATGGC

Table S2 The primer sequences for the identification of transformants.

Number	Primer name	Primer sequence (5′ to 3′)	Notes
1	ITS1	TCCGTAGGTGAACCTGCGG	Taxonomic identification of transformants
2	ITS4	TCCTCCGCTTATTGATATGC	
3	LPU-F	CCACCAGCTTATATACCTTAGCAGGAG	To eliminate DNA contamination from *Agrobacterium tumefaciens*
4	LPU-R	GTAACCATGCATGGTTGCCTAGTG	
5	RPU-R	TTGGTCAAGTCCTGGTCGTCGG	
6	LPUS-R	CAACGATTCCAGTACTCTTGGTCC	

Table S3 The primer sequences for mhiTAIL-PCR.

Number	Primer name	Primer sequence (5′ to 3′)	Notes
1	mLAD1	GCTCACGATGGACTGCTGAGTGGCACCTGVNVNNGGAA	The arbitrary degenerate primers
2	mLAD2	GCTCACGATGGACTGCTGAGTGGCACCTGBNBNNCCTT	
3	mLAD3	GCTCACGATGGACTGCTGAGTGGCACCTGHNHNNAACC	
4	mLAD4	GCTCACGATGGACTGCTGAGTGGCACCTGDNDNNTTGG	
5	AC0	GAGCTCACGATGGACTGC	The specific primers capable of binding to mLADs
6	AC1	CGATGGACTGCTGAGT	
7	RB-0a	CCCAGAATGCACAGGTACACTTG	The specific primers that can bind to the T-DNA region
8	RB-1a	CGATGGACTGCTGAGTGGCACCTGTGTAAGCGCCCACTCCACAT	
9	RB-2a	CCAACTTAATCGCCTTGCAGCACAT	
10	LB-0s	TAGGCACTCTTTCTGACCGCAAC	
11	LB-1s	TACGCGGAGTTTCGACTTACC	
12	LB-2u	CCCCGAATTAATTCGGCGTTAATTCAGTAC	

Annotation: V=A,G,C; H=A,C,T; D=A,G,T; B=G,C,T; N=A,G,C,T

Table S4 The GPD sequence information from 82 species was used for constructing the evolutionary tree in *S. rugosoannulata*.

Scientific Name	Taxid	Max Score	Total Score	Query Cover	E value	Per. ident	Acc. Len	Accession	Description
Hypholoma sublateritium FD-334 SS-4	945553	621	621	100%	0	92.01	338	KJA17846.1	hypothetical protein HYPSUDRAFT_45836 [Hypholoma sublateritium FD-334 SS-4]
Psilocybe cyanescens	93625	609	609	100%	0	90.24	338	PPQ70921.1	hypothetical protein CVT25_004765 [Psilocybe cyanescens]
Agrocybe chaxingu	84603	602	602	100%	0	88.46	338	KAJ3510358.1	hypothetical protein NLJ89_g4726 [Agrocybe chaxingu]
Cyclocybe aegerita	1973307	601	601	100%	0	88.17	338	CAA7262662.1	unnamed protein product [Cyclocybe aegerita]
Crassisporium funariophilum	1954473	601	601	100%	0	88.17	338	KAF8154126.1	glyceraldehyde 3-phosphate dehydrogenase [Crassisporium funariophilum]
Thelephora ganbajun	370292	596	596	99%	0	86.27	336	KAF9649541.1	glyceraldehyde-3-phosphate dehydrogenase [Thelephora ganbajun]
Psilocybe cubensis	181762	596	596	100%	0	86.65	359	XP_047744562.1	Glyceraldehyde-3-phosphate dehydrogenase [Psilocybe cubensis]
Tulosesus angulatus	980116	595	595	100%	0	85.5	338	KAF6750766.1	glyceraldehyde-3-phosphate dehydrogenase [Tulosesus angulatus]
Candolleomyces efflorescens	2917767	593	593	100%	0	84.62	338	KAJ2914888.1	hypothetical protein MD484_g5518, partial [Candolleomyces efflorescens]
Flammula alnicola	109635	593	593	100%	0	86.98	357	KAF8951277.1	glyceraldehyde 3-phosphate dehydrogenase [Flammula alnicola]
Gymnopilus junonius	109634	592	592	100%	0	85.26	348	KAF8901011.1	glyceraldehyde 3-phosphate dehydrogenase [Gymnopilus junonius]
Mucidula mucida	139077	590	590	100%	0	83.73	338	KAF8905629.1	glyceraldehyde-3-phosphate dehydrogenase [Mucidula mucida]
Cyathus striatus	68777	590	590	99%	0	87.8	336	KAF8999804.1	glyceraldehyde 3-phosphate dehydrogenase [Cyathus striatus]
Neolentinus lepideus HHB14362 ss-1	1314782	590	590	99%	0	85.33	336	KZT23015.1	glyceraldehyde-3-phosphate dehydrogenase [Neolentinus lepideus HHB14362 ss-1]
Cyathus bulleri	184115	589	589	97%	0	89.02	338	UDP83121.1	glyceraldehyde-3-phosphate dehydrogenase [Cyathus bulleri]
Tulosesus angulatus	980116	588	588	100%	0	83.05	348	KAF5326526.1	hypothetical protein D9611_000985 [Tulosesus angulatus]
Crucibulum laeve	68775	588	588	97%	0	90.55	337	TFK36503.1	glyceraldehyde 3-phosphate dehydrogenase [Crucibulum laeve]

Hymenopellis radicata	937743	587	587	100%	0	83.14	338	KAF9034471.1	glyceraldehyde-3-phosphate dehydrogenase [Hymenopellis radicata]
Agrocybe pediades	84607	587	587	100%	0	85.8	345	KAF9562895.1	glyceraldehyde 3-phosphate dehydrogenase [Agrocybe pediades]
Agrocybe pediades	84607	586	586	100%	0	85.55	346	KAF4611543.1	hypothetical protein D9613_003857 [Agrocybe pediades]
Cyathus stercoreus	181520	586	586	99%	0	87.32	340	BAN13558.1	glyceraldehyde-3-phosphate dehydrogenase [Cyathus stercoreus]
Mycena rebaudengoi	1045383	586	586	99%	0	85.59	335	KAJ7270909.1	glyceraldehyde-3-phosphate dehydrogenase [Mycena rebaudengoi]
Galerina marginata CBS 339.88	685588	586	586	97%	0	88.45	354	KDR78348.1	hypothetical protein GALMADRAFT_245488 [Galerina marginata CBS 339.88]
Armillaria mellea	47429	585	585	100%	0	83.48	339	KAK0189631.1	glyceraldehyde 3-phosphate dehydrogenase [Armillaria mellea]
Laccaria bicolor S238N-H82	486041	585	585	100%	0	85.8	338	XP_001887370.1	glyceraldehyde 3-phosphate dehydrogenase [Laccaria bicolor S238N-H82]
Laccaria bicolor S238N-H82	486041	585	585	100%	0	86.39	338	XP_001879716.1	glyceraldehyde 3-phosphate dehydrogenase [Laccaria bicolor S238N-H82]
Panaeolus papilionaceus	330517	585	585	97%	0	89.36	339	KAF9054199.1	glyceraldehyde 3-phosphate dehydrogenase [Panaeolus papilionaceus]
Pholiota conissans	109636	583	583	100%	0	87.28	334	KAF9480267.1	glyceraldehyde 3-phosphate dehydrogenase [Pholiota conissans]
Desarmillaria tabescens	1929756	582	582	100%	0	83.19	339	XP_060326328.1	glyceraldehyde 3-phosphate dehydrogenase [Desarmillaria tabescens]
Desarmillaria tabescens	1929756	582	582	100%	0	82.89	339	CAF74786.1	glyceraldehyde 3-phosphate dehydrogenase [Desarmillaria tabescens]
Mycena chlorophos	658473	582	582	99%	0	86.57	336	KAF7320882.1	Glyceraldehyde-3-phosphate dehydrogenase [Mycena chlorophos]
Cyathus bulleri	184115	582	582	97%	0	88.72	337	UES62894.1	glyceraldehyde-3-phosphate dehydrogenase [Cyathus bulleri]
Mycena polygramma	230801	582	582	99%	0	87.39	335	KAJ7602512.1	glyceraldehyde 3-phosphate dehydrogenase [Mycena polygramma]
Heliocybe sulcata	5364	581	581	99%	0	84.08	335	TFK48245.1	glyceraldehyde-3-phosphate dehydrogenase [Heliocybe sulcata]
Armillaria luteobubalina	153913	581	581	100%	0	82.89	339	KAK0480498.1	glyceraldehyde 3-phosphate dehydrogenase [Armillaria luteobubalina]
Mycena haematopus	230807	580	580	99%	0	83.83	336	KAJ7270768.1	glyceraldehyde-3-phosphate dehydrogenase [Mycena haematopus]
Armillaria nabsnona	64368	580	580	100%	0	82.6	339	KAK0236991.1	glyceraldehyde 3-phosphate dehydrogenase [Armillaria nabsnona]
Mycena vitilis	1033	578	578	99%	0	87.09	335	KAJ64689	glyceraldehyde-3-phosphate dehydrogenase [Mycena vitilis]

	281							94.1	
Phlegmacium glaucopus	171480	578	578	100%	0	84.32	338	KAF8797580.1	glyceraldehyde 3-phosphate dehydrogenase [Phlegmacium glaucopus]
Agaricus bisporus var. bisporus H97	936046	578	578	100%	0	81.07	338	XP_006455566.1	glyceraldehyde-3-phosphate dehydrogenase 2 [Agaricus bisporus var. bisporus H97]
Mycena rosella	1033263	577	577	99%	0	86.79	335	KAJ7686772.1	glyceraldehyde-3-phosphate dehydrogenase [Mycena rosella]
Amanita brunnescens Koide BX004	1457509	577	577	100%	0	81.95	358	KAF8727788.1	hypothetical protein AX14_007001 [Amanita brunnescens Koide BX004]
Antrodiella citrinella	2447956	577	577	99%	0	81.85	336	THH30455.1	hypothetical protein EUX98_g3735 [Antrodiella citrinella]
Agaricus bisporus var. burnettii JB137-S8	597362	576	576	100%	0	80.77	338	XP_007332717.1	uncharacterized protein AGABI1DRAFT_115671 [Agaricus bisporus var. burnettii JB137-S8]
Pleurotus ostreatus	5322	576	576	99%	0	85.67	336	KAJ8690257.1	hypothetical protein PTI98_011702 [Pleurotus ostreatus]
Agaricus subrufescens	87252	576	576	100%	0	79.59	338	QND78268.1	glyceraldehyde-3-phosphate dehydrogenase [Agaricus subrufescens]
Candolleomyces eurysporus	2828524	576	576	99%	0	86.83	336	KAJ2932010.1	hypothetical protein H1R20_g5085, partial [Candolleomyces eurysporus]
Leucoagaricus meleagris	201219	575	575	99%	0	80.36	339	ABU87583.1	glyceraldehyde-3-phosphate dehydrogenase [Leucoagaricus meleagris]
Desarmillaria ectypa	47430	575	575	100%	0	82.89	338	KAK0200875.1	glyceraldehyde 3-phosphate dehydrogenase [Desarmillaria ectypa]
Coprinopsis cinerea	5346	575	575	100%	0	81.95	340	BAC75713.1	glyceraldehyde-3-phosphate dehydrogenase [Coprinopsis cinerea]
Pleurotus eryngii	5323	575	575	99%	0	85.37	336	KAF9489890.1	glyceraldehyde 3-phosphate dehydrogenase [Pleurotus eryngii]
Pleurotus pulmonarius	28995	575	575	99%	0	85.59	342	KAL4255315.1	Glyceraldehyde-3-phosphate dehydrogenase [Pleurotus pulmonarius]
Coprinopsis cinerea okayama7#130	240176	575	575	100%	0	85.8	339	XP_001834159.2	glyceraldehyde-3-phosphate dehydrogenase [Coprinopsis cinerea okayama7#130]
Coprinellus micaceus	71717	574	574	100%	0	85.8	338	TEB36602.1	glyceraldehyde-3-phosphate dehydrogenase [Coprinellus micaceus]
Mycena galericulata	71905	574	574	99%	0	85.89	335	KAJ7452142.1	glyceraldehyde 3-phosphate dehydrogenase [Mycena galericulata]
Cutaneotrichosporon cavernicola	279322	573	573	99%	0	82.34	337	XP_060457007.1	uncharacterized protein CcaverHIS019_0405620 [Cutaneotrichosporon cavernicola]
Amanita strobiliformis	67730	573	573	99%	0	81.25	337	AFU72283.1	glyceraldehyde-3-phosphate dehydrogenase [Amanita strobiliformis]
Tricholomella constricta	117010	573	573	99%	0	82.93	337	KAF5376072.1	hypothetical protein D9615_007715 [Tricholomella constricta]

Clavulina sp. PMI_390	1486700	573	573	99%	0	81.79	336	KAF8317871.1	glyceraldehyde-3-phosphate dehydrogenase [Clavulina sp. PMI_390]
Mycena filopes	858877	573	573	99%	0	85.59	335	KAJ7160928.1	glyceraldehyde-3-phosphate dehydrogenase [Mycena filopes]
Coniophora puteana RWD-64-598 SS2	741705	572	572	99%	0	82.09	346	XP_007772938.1	glyceraldehyde-3-phosphate dehydrogenase [Coniophora puteana RWD-64-598 SS2]
Candolleomyces aberdarensis	2316362	572	572	99%	0	86.23	336	RXW20144.1	hypothetical protein EST38_g5709 [Candolleomyces aberdarensis]
Mycena alexandri	1745969	571	571	99%	0	85.29	335	KAJ7042770.1	glyceraldehyde-3-phosphate dehydrogenase [Mycena alexandri]
Laetiporus sulphureus 93-53	1314785	571	571	99%	0	81.14	337	XP_040761042.1	glyceraldehyde-3-phosphate dehydrogenase [Laetiporus sulphureus 93-53]
Trametes pubescens	154538	571	571	99%	0	82.28	359	OJT05012.1	Glyceraldehyde-3-phosphate dehydrogenase [Trametes pubescens]
Cutaneotrichosporon spelunceum	1672016	571	571	99%	0	81.85	337	GMK59319.1	hypothetical protein CspeluHIS016_0703340 [Cutaneotrichosporon spelunceum]
Schizophyllum amplum	97359	571	571	99%	0	81.74	337	TRM60041.1	glyceraldehyde-3-phosphate dehydrogenase [Schizophyllum amplum]
Psilocybe cf. subviscida	2480587	571	571	100%	0	83.98	342	KAF5314478.1	hypothetical protein D9619_011765 [Psilocybe cf. subviscida]
Mycena sp. CBHHK59/15	1940295	570	570	99%	0	85.89	335	KAJ6630130.1	glyceraldehyde-3-phosphate dehydrogenase [Mycena sp. CBHHK59/15]
Laccaria amethystina LaAM-08-1	1095629	570	570	98%	0	85.15	332	KIJ92349.1	hypothetical protein K443DRAFT_113743, partial [Laccaria amethystina LaAM-08-1]
Trametes elegans	526244	569	569	98%	0	81.33	337	KAI0776770.1	glyceraldehyde-3-phosphate dehydrogenase [Trametes elegans]
Cutaneotrichosporon sp. HIS471	3040995	569	569	99%	0	81.44	337	BEJ14780.1	hypothetical protein CspHIS471_0405470 [Cutaneotrichosporon sp. HIS471]
Mycena latifolia	1033247	569	569	99%	0	85.59	335	KAJ7456881.1	glyceraldehyde 3-phosphate dehydrogenase [Mycena latifolia]
Mycena galericulata	71905	569	569	99%	0	85.29	335	KAJ7475503.1	glyceraldehyde 3-phosphate dehydrogenase [Mycena galericulata]
Trametes versicolor	5325	569	569	99%	0	81.98	341	BDI62033.1	glyceraldehyde-3-phosphate dehydrogenase [Trametes versicolor]
Marasmius oreades	181124	568	568	99%	0	81.25	336	XP_043003900.1	uncharacterized protein E1B28_013395 [Marasmius oreades]
Pleurotus ostreatus	5322	568	568	99%	0	83.58	336	BAL44217.1	glyceraldehyde 3-phosphate dehydrogenase [Pleurotus ostreatus]
Pleurotus ostreatus	5322	568	568	99%	0	83.58	338	BAL44215.1	glyceraldehyde 3-phosphate dehydrogenase [Pleurotus ostreatus]
Pleurotus ostreatus	5322	568	568	99%	0	83.28	336	KAJ86902	hypothetical protein PTI98_011666 [Pleurotus ostreatus]

								14.1	
Hypsizygus marmoreus	39966	568	568	99%	0	85.93	338	AER27744.1	glyceraldehyde-3-phosphate dehydrogenase [Hypsizygus marmoreus]
Pleurotus ostreatus	5322	568	568	99%	0	82.99	342	KAJ8690215.1	hypothetical protein PTI98_011666 [Pleurotus ostreatus]
Mycena chlorophos	658473	568	568	99%	0	85.03	337	GAT50258.1	glyceraldehyde-3-phosphate dehydrogenase [Mycena chlorophos]
Mycena sanguinolenta	230812	568	568	99%	0	85.29	335	KAJ6463393.1	glyceraldehyde-3-phosphate dehydrogenase [Mycena sanguinolenta]
Leucoagaricus leucothites	201217	568	568	100%	0	81.36	338	KAF5353208.1	hypothetical protein D9756_007853 [Leucocoprinus leucothites]
Flammulina velutipes	38945	568	568	100%	0	80.12	339	AAQ08201.1	glyceraldehyde-3-phosphate dehydrogenase [Flammulina velutipes]
Pleurotus ostreatus	5322	567	567	99%	0	83.28	336	ACY35994.1	glyceraldehyde-3-phosphate dehydrogenase [Pleurotus ostreatus]
Pleurotus ostreatus PC15	1137138	567	567	99%	0	83.28	342	KDQ24107.1	hypothetical protein PLEOSDRAFT_1090672 [Pleurotus ostreatus PC15]
Moniliophthora roreri MCA 2997	1381753	567	567	99%	0	80.06	336	ESK93893.1	glyceraldehyde-3-phosphate dehydrogenase [Moniliophthora roreri MCA 2997]
Serendipita sp. 411	2600216	567	567	98%	0	81.93	336	KAG8849822.1	hypothetical protein FRB91_009593 [Serendipita sp. 411]
Cristinia sonorae	1940300	567	567	96%	0	84.36	337	KAH8079837.1	glyceraldehyde-3-phosphate dehydrogenase [Cristinia sonorae]
Trametes versicolor FP-101664 SS1	717944	567	567	99%	0	81.68	341	XP_008040088.1	glyceraldehyde-3-phosphate dehydrogenase [Trametes versicolor FP-101664 SS1]
Auricularia polytricha	29893	566	566	100%	0	80.53	339	AIB06356.1	glyceraldehyde-3-phosphate dehydrogenase [Auricularia polytricha]
Phellopilus nigrolimitatus	127956	566	566	100%	0	81.07	339	KAH8112048.1	glyceraldehyde 3-phosphate dehydrogenase [Phellopilus nigrolimitatus]
Pleurotus citrinopileatus	98342	566	566	99%	0	84.78	336	UOW59936.1	glyceraldehyde-3-phosphate dehydrogenase [Pleurotus citrinopileatus]

Table S5 The SDI sequence information from 87 species was used for constructing the evolutionary tree in *S. rugosoannulata*.

Scientific Name	Taxid	Max Score	Total Score	Query Cover	E value	Per. ident	Acc. Len	Accession	Description
Hypholoma sublateritium FD-334 SS-4	945553	533	533	100%	0	92.88	267	KJA18291.1	hypothetical protein HYPSUDRAFT_205533 [Hypholoma sublateritium FD-334 SS-4]
Galerina marginata CBS 339.88	685588	517	517	100%	0	89.26	270	KDR70285.1	hypothetical protein GALMADRAFT_255190 [Galerina marginata CBS 339.88]
Pholiota molesta	2066989	516	516	100%	0	91.48	270	KAF8178898.1	succinate dehydrogenase iron-sulfur protein subunit [Pholiota molesta]
Flammula alnicola	109635	515	515	100%	0	90	270	KAF8962070.1	succinate dehydrogenase iron-sulfur subunit [Flammula alnicola]
Pholiota conissans	109636	509	509	100%	0	89.63	270	KAF9481759.1	succinate dehydrogenase iron-sulfur protein subunit [Pholiota conissans]
Agrocybe pediades	84607	508	508	100%	0	88.15	270	KAF9552431.1	succinate dehydrogenase iron-sulfur subunit [Agrocybe pediades]
Panaeolus papilionaceus	330517	507	507	100%	2E-180	88.15	270	KAF9042960.1	succinate dehydrogenase iron-sulfur subunit [Panaeolus papilionaceus]
Cyclocybe aegerita	1973307	504	504	100%	2E-179	88.76	266	APA05128.1	succinate dehydrogenase iron-sulfur subunit [Cyclocybe aegerita]
Psilocybe cubensis	181762	504	504	100%	2E-179	87.04	270	XP_047743913.1	succinate dehydrogenase complex, subunit B [Psilocybe cubensis]
Phlegmacium glaucopus	171480	504	504	100%	4E-179	87.04	270	KAF8806746.1	succinate dehydrogenase iron-sulfur subunit [Phlegmacium glaucopus]
Psilocybe cyanescens	93625	503	503	100%	2E-178	86.67	270	PPQ78467.1	hypothetical protein CVT25_011862 [Psilocybe cyanescens]
Pholiota nameko	61267	503	503	100%	2E-178	87.82	271	BAH30231.1	succinate dehydrogenase iron-sulfur protein subunit [Pholiota nameko]
Agrocybe chaxingu	84603	502	502	100%	3E-178	88.01	266	KAJ3499919.1	hypothetical protein NLJ89_g10016 [Agrocybe chaxingu]
Panaeolus cyanescens	181874	502	502	100%	4E-178	86.55	275	PPR00683.1	hypothetical protein CVT24_000971 [Panaeolus cyanescens]
Crassisporium funariophilum	1954473	501	501	100%	8E-178	86.45	273	KAF8154463.1	iron-sulphar subunit protein of succinate dehydrogenase [Crassisporium funariophilum]
Gymnopilus junonius	109634	501	501	97%	8E-178	89.96	272	KAF8907616.1	succinate dehydrogenase iron-sulfur protein subunit [Gymnopilus junonius]
Hebeloma cylindrosporum	76867	498	498	100%	1E-176	86.3	270	KIM43854.1	hypothetical protein M413DRAFT_390042 [Hebeloma cylindrosporum]

Hebeloma cylindrosporum	76867	498	498	100%	2E-176	86.3	270	CAT00089.1	succinate dehydrogenase iron-sulfur subunit [Hebeloma cylindrosporum]
Pisolithus microcarpus	178872	494	494	99%	7E-175	86.19	277	KAI6020651.1	iron-sulfur protein subunit of succinate dehydrogenase, Sdh2p [Pisolithus microcarpus]
Gymnopilus dilepis	231916	493	493	100%	1E-174	85.98	270	PPR04631.1	hypothetical protein CVT26_015010 [Gymnopilus dilepis]
Pisolithus albus	178870	491	491	99%	1E-173	85.45	277	KAI5992888.1	iron-sulfur protein subunit of succinate dehydrogenase, Sdh2p [Pisolithus albus]
Termitomyces sp. T159_Od127	2053459	490	490	100%	1E-173	85.13	269	KAG6898631.1	succinate dehydrogenase complex, subunit B [Termitomyces sp. T159_Od127]
Pisolithus albus	178870	491	491	99%	1E-173	85.45	277	KAI5997400.1	iron-sulfur protein subunit of succinate dehydrogenase, Sdh2p [Pisolithus albus]
Tricholoma furcatifolium	2724999	489	489	100%	5E-173	85.13	269	KAG6829350.1	succinate dehydrogenase complex, subunit B [Tricholoma furcatifolium]
Pisolithus croceorrhizus	2060934	489	489	99%	6E-173	85.07	277	KAI6104738.1	iron-sulphar subunit protein of succinate dehydrogenase [Pisolithus croceorrhizus]
Hydnomerulius pinastri MD-312	994086	488	488	100%	2E-172	81.45	275	KIJ60855.1	hypothetical protein HYDPIDRAFT_116720 [Hydnomerulius pinastri MD-312]
Pisolithus marmoratus	178871	486	486	100%	8E-172	84.27	277	KAI6043792.1	iron-sulphar subunit protein of succinate dehydrogenase [Pisolithus marmoratus]
Cyathus striatus	68777	485	485	100%	1E-171	84.56	271	KAF9002003.1	iron-sulfur subunit protein of succinate dehydrogenase [Cyathus striatus]
Tephrocybe sp. NHM501043	2724996	484	484	100%	2E-171	84.76	269	KAG6855792.1	succinate dehydrogenase complex, subunit B [Tephrocybe sp. NHM501043]
Sphagnurus paluster	117069	483	483	100%	8E-171	83.52	273	KAG5651133.1	succinate dehydrogenase complex, subunit B [Sphagnurus paluster]
Melanogaster broomeanus	2655010	482	482	100%	2E-170	81.09	275	KAF9236714.1	iron-sulfur protein subunit of succinate dehydrogenase, Sdh2p [Melanogaster broomeanus]
Laccaria bicolor S238N-H82	486041	481	481	96%	3E-170	87.06	263	XP_001876249.1	uncharacterized protein LACBIDRAFT_182791 [Laccaria bicolor S238N-H82]
Asterophora parasitica	117018	482	482	99%	3E-170	84.09	271	KAG5644429.1	succinate dehydrogenase complex, subunit B [Asterophora parasitica]
Termitomyces sp. T32_za158	2053458	482	482	100%	4E-170	82.12	274	KAG6876316.1	succinate dehydrogenase complex, subunit B [Termitomyces sp. T32_za158]
Paxillus involutus ATCC 200175	664439	481	481	100%	5E-170	82.18	275	KIJ08477.1	succinate dehydrogenase [Paxillus involutus ATCC 200175]
Arthromyces matolae	530044	480	480	97%	1E-169	86.1	268	KAG6819589.1	succinate dehydrogenase complex, subunit B [Arthromyces matolae]
Leucogyrophana mollusca	85980	480	480	100%	1E-169	83.39	271	KAH7929185.1	iron-sulfur subunit protein of succinate dehydrogenase [Leucogyrophana mollusca]
Gyrodon lividus	85971	480	480	100%	2E-	80.36	275	KAF92227	iron-sulphar subunit protein of succinate dehydrogenase [Gyrodon

					169			78.1	lividus]
Imleria badia	36058	479	479	100%	2E-169	84.64	266	KAF8554967.1	succinate dehydrogenase and fumarate r [Imleria badia]
Volvariella volvacea WC 439	1457512	479	479	100%	3E-169	82.05	273	KAF8652503.1	hypothetical protein AX16_004376 [Volvariella volvacea WC 439]
Collybia nuda	64659	479	479	95%	3E-169	87.75	272	KAF9457439.1	iron-sulphar subunit protein of succinate dehydrogenase [Collybia nuda]
Laccaria amethystina LaAM-08-1	1095629	479	479	96%	3E-169	86.67	263	KIK01904.1	hypothetical protein K443DRAFT_678046 [Laccaria amethystina LaAM-08-1]
Fistulina hepatica ATCC 64428	1128425	479	479	99%	3E-169	85.66	271	KIY45193.1	iron-sulfur subunit protein of succinate dehydrogenase [Fistulina hepatica ATCC 64428]
Termitomyces sp. Mn162	2053456	479	479	100%	3E-169	82.35	272	KAG5332839.1	succinate dehydrogenase complex, subunit B [Termitomyces sp. Mn162]
Chiua virens	1914213	479	479	100%	4E-169	82.4	267	KAG9309713.1	iron-sulphar subunit protein of succinate dehydrogenase [Chiua virens]
Butyriboletus roseoflavus	1325616	479	479	99%	4E-169	83.77	275	KAG8221393.1	iron-sulphar subunit protein of succinate dehydrogenase [Butyriboletus roseoflavus]
Boletus reticuloceps	495285	479	479	100%	5E-169	83.03	271	KAG6370483.1	iron-sulphar subunit protein of succinate dehydrogenase [Boletus reticuloceps]
Athelia sp. TMB	2748771	479	479	100%	5E-169	83.08	272	KAF7971598.1	hypothetical protein HWV62_20776 [Athelia sp. TMB]
Piloderma croceum F 1598	765440	478	478	95%	6E-169	87.35	272	KIM80193.1	hypothetical protein PILCRDRAFT_822712 [Piloderma croceum F 1598]
Scleroderma yunnanense	1163567	478	478	99%	7E-169	83.21	276	KAL4063589.1	alpha-helical ferredoxin [Scleroderma yunnanense]
Paxillus ammoniavirescens	1132860	478	478	100%	1E-168	81.09	275	KAF8838246.1	iron-sulphar subunit protein of succinate dehydrogenase [Paxillus ammoniavirescens]
Dendrothele bispora CBS 962.96	1314807	477	477	99%	2E-168	84.33	269	THV05036.1	iron-sulphar subunit protein of succinate dehydrogenase [Dendrothele bispora CBS 962.96]
Boletus edulis BED1	1328754	477	477	100%	2E-168	83.39	271	KAF8436474.1	iron-sulfur subunit protein of succinate dehydrogenase [Boletus edulis BED1]
Serpula lacrymans var. lacrymans S7.9	578457	477	477	99%	2E-168	81.99	279	XP_007323431.1	iron-sulfur protein subunit of succinate dehydrogenase, Sdh2p [Serpula lacrymans var. lacrymans S7.9]
Paxillus rubicundulus Ve08.2h10	930991	477	477	100%	3E-168	81.48	275	KIK82102.1	hypothetical protein PAXRUDRAFT_832396 [Paxillus rubicundulus Ve08.2h10]
Hohenbuehelia grisea	104357	476	476	100%	5E-168	83.03	271	KAL0945256.1	hypothetical protein HGRIS_000766 [Hohenbuehelia grisea]
Termitomyces sp. Mi166#008	2053460	476	476	100%	5E-168	83.15	265	KAG6871684.1	succinate dehydrogenase complex, subunit B [Termitomyces sp. Mi166#008]
Scleroderma citrinum Foug A	1036808	475	475	99%	1E-167	82.46	276	KIM66545.1	hypothetical protein SCLCIDRAFT_110407 [Scleroderma citrinum Foug A]

Plicaturopsis crispa FD-325 SS-3	944288	475	475	100%	2E-167	83.39	270	KII85656.1	hypothetical protein PLICRDRAFT_116306 [Plicaturopsis crispa FD-325 SS-3]
Leucocoprinus birnbaumii	56174	474	474	98%	3E-167	84.73	269	KAJ3575397.1	hypothetical protein NP233_g1123 [Leucocoprinus birnbaumii]
Lentinula raphanica	153919	474	474	96%	4E-167	86.33	272	KAJ3755233.1	iron-sulphar subunit protein of succinate dehydrogenase [Lentinula raphanica]
Lentinula raphanica	153919	474	474	99%	5E-167	83.03	272	KAJ3724499.1	iron-sulphar subunit protein of succinate dehydrogenase [Lentinula raphanica]
Coniophora puteana RWD-64-598 SS2	741705	474	474	100%	5E-167	81.75	274	XP_007774143.1	iron-sulphar subunit protein of succinate dehydrogenase [Coniophora puteana RWD-64-598 SS2]
Lentinula edodes	5353	473	473	99%	6E-167	82.66	272	KAJ4467885.1	iron-sulphar subunit protein of succinate dehydrogenase [Lentinula edodes]
Hygrophoropsis aurantiaca	72124	473	473	100%	6E-167	80.81	271	KAH7915501.1	iron-sulfur protein subunit of succinate dehydrogenase, Sdh2p [Hygrophoropsis aurantiaca]
Lentinula raphanica	153919	473	473	96%	7E-167	86.33	272	KAJ3820093.1	iron-sulphar subunit protein of succinate dehydrogenase [Lentinula raphanica]
Boletus coccyginus	1758874	473	473	99%	7E-167	82.95	264	KAI9462567.1	iron-sulfur protein subunit of succinate dehydrogenase, Sdh2p [Boletus coccyginus]
Amanita brunnescens Koide BX004	1457509	473	473	95%	8E-167	86.96	268	KAF8729245.1	hypothetical protein AX14_006291 [Amanita brunnescens Koide BX004]
Tricholoma matsutake 945	1095628	473	473	95%	8E-167	87.75	263	KAF8238630.1	succinate dehydrogenase iron-sulfur subunit [Tricholoma matsutake 945]
Amylostereum chailletii	55340	473	473	100%	9E-167	82.77	273	KAI0316606.1	succinate dehydrogenase iron-sulfur subunit [Amylostereum chailletii]
Paxillus ammoniavirescens	1132860	473	473	100%	1E-166	79.27	275	KAF8837587.1	iron-sulphar subunit protein of succinate dehydrogenase, partial [Paxillus ammoniavirescens]
Flammulina velutipes	38945	473	473	100%	1E-166	82.02	267	AVL25507.1	succinate dehydrogenase iron-sulfur subunit [Flammulina velutipes]
Pisolithus sp. B1	2614595	472	472	93%	1E-166	87.9	260	KAI6137392.1	succinate dehydrogenase iron-sulfur subunit [Pisolithus sp. B1]
Lentinula edodes	5353	473	473	99%	1E-166	82.29	272	KAJ3882167.1	iron-sulphar subunit protein of succinate dehydrogenase [Lentinula edodes]
Mycena floridula	1033242	473	473	95%	2E-166	86.67	277	KAJ7593747.1	iron-sulphar subunit protein of succinate dehydrogenase [Mycena floridula]
Lentinula novae-zelandiae	42442	472	472	99%	2E-166	82.29	272	KAJ3865448.1	iron-sulphar subunit protein of succinate dehydrogenase [Lentinula novae-zelandiae]
Schizophyllum fasciatum	160053	472	472	100%	2E-166	81.92	271	KAL1745279.1	iron-sulfur subunit protein of succinate dehydrogenase [Schizophyllum fasciatum]
Lentinula edodes	5353	472	472	99%	2E-166	82.66	272	KAJ3910028.1	iron-sulphar subunit protein of succinate dehydrogenase [Lentinula edodes]
Moniliophthora roreri	13817	472	472	100%	2E-	80.87	277	ESK97184	succinate dehydrogenase iron-sulfur subunit [Moniliophthora

MCA 2997	53				166			.1	roreri MCA 2997]
Heterobasidion irregulare TC 32-1	747525	472	472	100%	2E-166	82.66	271	XP_009552678.1	uncharacterized protein HETIRDRAFT_481926 [Heterobasidion irregulare TC 32-1]
Lentinula lateritia	40482	472	472	99%	2E-166	82.29	272	KAJ3937492.1	MAG: iron-sulphar subunit protein of succinate dehydrogenase [Lentinula lateritia]
Lentinula guzmanii	2804957	471	471	99%	4E-166	84.03	272	KAJ3735699.1	iron-sulphar subunit protein of succinate dehydrogenase [Lentinula guzmanii]
Hymenopellis radicata	937743	471	471	96%	5E-166	85.94	263	KAF9019499.1	succinate dehydrogenase and fumarate r [Hymenopellis radicata]
Pluteus cervinus	181527	471	471	100%	6E-166	84.5	271	TFK66484.1	iron-sulphar subunit protein of succinate dehydrogenase [Pluteus cervinus]
Lentinula detonsa	2804962	471	471	99%	7E-166	84.41	272	KAJ3989094.1	iron-sulphar subunit protein of succinate dehydrogenase [Lentinula detonsa]
Scleroderma citrinum Foug A	1036808	471	471	97%	7E-166	83.72	276	KIM62824.1	hypothetical protein SCLCIDRAFT_118652 [Scleroderma citrinum Foug A]
Amanita polypyramis BW_CC	1457511	471	471	99%	7E-166	83.77	272	KAF8635136.1	hypothetical protein AX15_000522 [Amanita polypyramis BW_CC]
Stereum hirsutum FP-91666 SS1	721885	470	470	100%	9E-166	81.62	271	XP_007306928.1	succinate dehydrogenase and fumarate r [Stereum hirsutum FP-91666 SS1]
Collybiopsis luxurians FD-317 M1	944289	470	470	99%	1E-165	81.92	272	KIK67418.1	hypothetical protein GYMLUDRAFT_93224 [Collybiopsis luxurians FD-317 M1]
Lentinula aff. lateritia	2804960	470	470	99%	1E-165	81.92	272	KAJ3815894.1	iron-sulphar subunit protein of succinate dehydrogenase [Lentinula aff. lateritia]
Cristinia sonorae	1940300	470	470	100%	1E-165	82.16	269	KAH8103283.1	succinate dehydrogenase and fumarate r [Cristinia sonorae]
Moniliophthora roreri	221103	470	470	100%	1E-165	80.14	277	KAI3618941.1	succinate dehydrogenase iron-sulfur subunit [Moniliophthora roreri]
Athelia psychrophila	1759441	470	470	100%	1E-165	81.95	271	KZP10275.1	succinate dehydrogenase and fumarate r [Athelia psychrophila]
Amanita inopinata Kibby_2008	1457513	469	469	95%	2E-165	85.77	274	KAF8623245.1	hypothetical protein AX17_007491 [Amanita inopinata Kibby_2008]
Blastosporella zonata	530045	469	469	96%	2E-165	83.66	269	KAG6865529.1	succinate dehydrogenase complex, subunit B [Blastosporella zonata]
